# Rewiring of jasmonate and phytochrome B signalling uncouples plant growth-defense tradeoffs

**DOI:** 10.1038/ncomms12570

**Published:** 2016-08-30

**Authors:** Marcelo L. Campos, Yuki Yoshida, Ian T. Major, Dalton de Oliveira Ferreira, Sarathi M. Weraduwage, John E. Froehlich, Brendan F. Johnson, David M. Kramer, Georg Jander, Thomas D. Sharkey, Gregg A. Howe

**Affiliations:** 1 Department of Energy-Plant Research Laboratory, East Lansing, Michigan 48824, USA; 2 Department of Biochemistry and Molecular Biology, Michigan State University, East Lansing, Michigan 48824, USA; 3 Boyce Thompson Institute for Plant Research, Ithaca, New York 14853, USA

## Abstract

Plants resist infection and herbivory with innate immune responses that are often associated with reduced growth. Despite the importance of growth-defense tradeoffs in shaping plant productivity in natural and agricultural ecosystems, the molecular mechanisms that link growth and immunity are poorly understood. Here, we demonstrate that growth-defense tradeoffs mediated by the hormone jasmonate are uncoupled in an *Arabidopsis* mutant (*jazQ phyB*) lacking a quintet of Jasmonate ZIM-domain transcriptional repressors and the photoreceptor phyB. Analysis of epistatic interactions between *jazQ* and *phyB* reveal that growth inhibition associated with enhanced anti-insect resistance is likely not caused by diversion of photoassimilates from growth to defense but rather by a conserved transcriptional network that is hardwired to attenuate growth upon activation of jasmonate signalling. The ability to unlock growth-defense tradeoffs through relief of transcription repression provides an approach to assemble functional plant traits in new and potentially useful ways.

## Abstract (web summary)

Plant immune responses are often associated with reduced growth. Here, the authors show that combining mutations in transcriptional repressors of the defense and light perception pathways can confer both robust growth and strong herbivore defense, demonstrating that growth-defense tradeoffs can be uncoupled.

## Graphical abstract

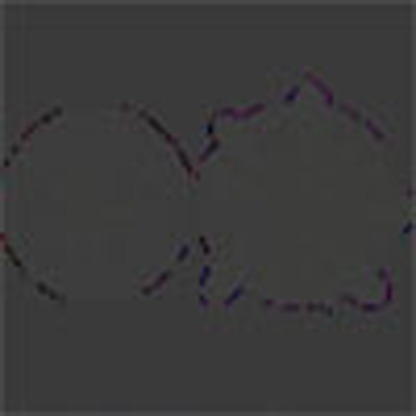

Plants continuously monitor external cues to tailor their growth, development and defensive capabilities in ways that optimize reproductive success, especially in environments where nutrients and light may be scarce. Theories to explain the costs and patterns of plant defense often invoke the existence of physiological tradeoffs that arise from allocation of limited resources to protective compounds and associated morphological structures^1^,^2^,^3^. Thus, a common interpretation of the ‘dilemma of plants to grow or defend'^3^ is that elevated expression of defense traits consumes metabolic resources at the expense of growth, whereas rapid growth, such as that triggered by shade light from competitors, diverts resources that could otherwise be invested in the defense arsenal. Although the concept of growth-defense tradeoffs is a major paradigm in ecological studies of plant resistance to herbivores and pathogens^1^,^2^,^3^,^4^, recent studies question the simplistic view that allocation of limited metabolic resources to one process necessarily reduces energetic expenditures in the other process^5^,^6^,^7^,^8^,^9^,^10^. These collective studies highlight the need to develop a more accurate conceptual framework for understanding how tradeoffs between growth and immunity optimize plant fitness in dynamic environments.

Here, we sought to exploit genetic tools in *Arabidopsis thaliana* to better understand how growth and defense are mechanistically integrated and to explicitly test whether the underlying signalling networks can be reconfigured to allow for simultaneous growth and defense. Our approach was based on the fact that defense responses mediated by the lipid-derived hormone jasmonate (JA) are associated with potent growth inhibition of leaves and roots^11^,^12^,^13^,^14^. We show that growth-defense tradeoffs are uncoupled in leaves of an *Arabidopsis* mutant (*jazQ phyB*) lacking five Jasmonate ZIM-domain (JAZ) transcriptional repressors and the photoreceptor phytochrome B (phyB). The concomitant robust growth and heightened anti-insect defense of *jazQ phyB* plants can be attributed, at least in part, to parallel activation of MYC and PIF transcription factors that in wild-type (WT) plants are repressed by JAZ and phyB, respectively. We further show that the capacity of *jazQ phyB* plants to grow and defend well simultaneously is associated with changes in leaf architecture and increased partitioning of carbon to leaf-area growth, but does not depend on increased leaf area-based photosynthesis. Thus, growth-defense antagonism in this system does not appear to be caused by constraints on the availability of metabolic resources that fuel growth and defensive processes but rather by a hormone-linked transcriptional network that is hardwired to restrict growth and upon activation of JA signalling. These collective findings highlight the importance of transcriptional repressor proteins in optimizing growth-defense balance, and further suggest that genetic modification of pathways that integrate defense and light signalling is a potential strategy to combine growth and defense traits in new ways.

## Results

### A *jaz* quintuple mutant exhibits constitutive JA responses

**Figure 1:**
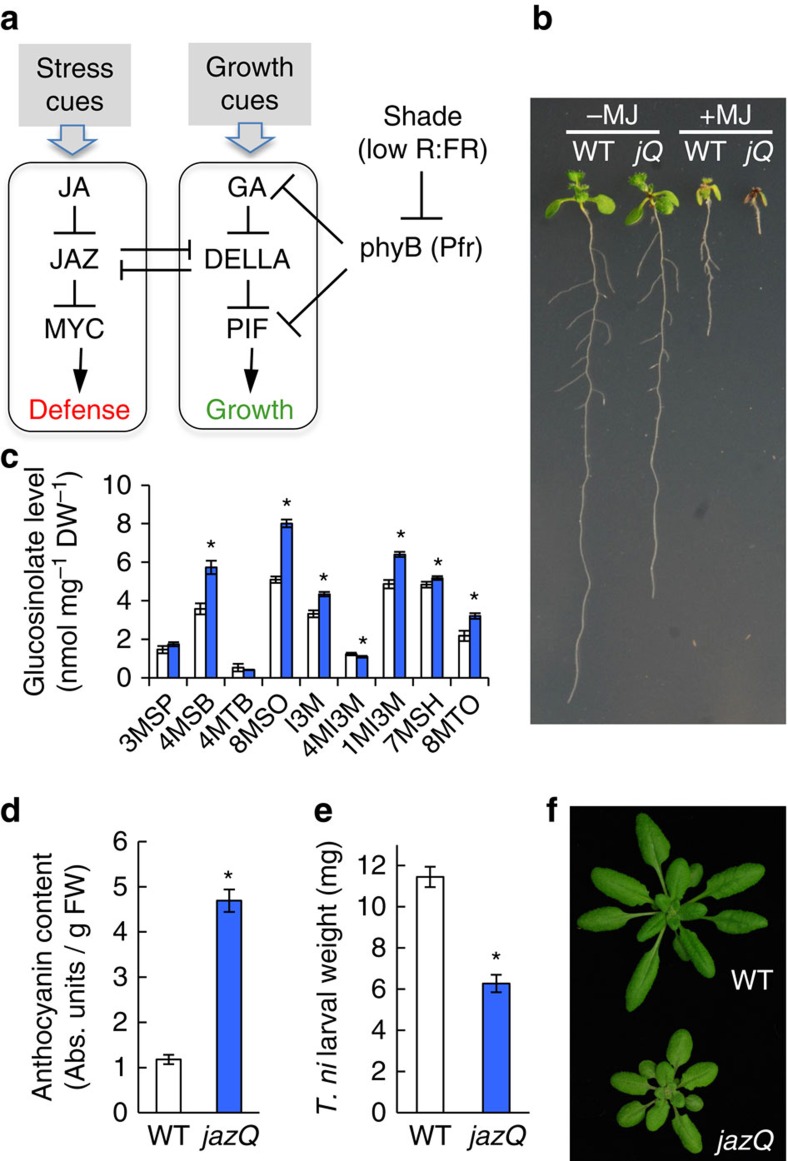
A *jaz* quintuple mutant exhibits reduced growth and enhanced defense. (**a**) Simple model of the JA-GA signalling network that governs growth and defense. (**b**) Photograph of WT and *jazQ* (*jQ*) seedlings grown in the absence or presence of 25 μM MeJA (MJ). (**c**) Accumulation of glucosinolates in WT (open bar) and *jazQ* (blue) seedlings. Compound abbreviations are listed in Methods section. (**d**) Anthocyanin accumulation in petioles of 4-week-old plants. (**e**) *Trichoplusia ni* weight after feeding on WT (33 larvae) and *jazQ* (38 larvae) plants for 10 days. (**f**) Photograph of 4-week-old soil-grown WT and *jazQ* plants. Data in all graphs represent the mean±s.e.m. of at least 10 biological replicates. Asterisks in **c**–**e** denote significant differences between WT and *jazQ* at *P*<0.05 (Student's *t*-test).

We devised a genetic screen to identify mutants of *Arabidopsis* that display enhanced JA-regulated defense against insect herbivory without an associated reduction in leaf growth. This screen was based on a signalling model^15^ predicting that removal of JAZ repressor proteins would constitutively activate anti-insect defense and inhibit growth of vegetative tissues (Fig. 1a). Such a phenotype has not been previously observed for *jaz* loss-of-function mutants of *Arabidopsis*, most likely because of functional redundancy between the 13 members of the *JAZ* gene family^16^. We thus developed an *Arabidopsis* line (*jaz* quintuple or *jazQ*) with T-DNA insertion mutations in five *JAZ* genes (*JAZ1*/*3*/*4*/*9*/*10*) (Supplementary Fig. 1). These JAZs were selected on the basis of their phylogenetic relationship, their demonstrated role in inhibiting various transcription factors (for example, MYCs) that execute defense responses, and their capacity to interact with DELLA proteins that antagonistically link JA signalling to gibberellic acid (GA)-mediated growth responses (Fig. 1a)^17^,^18^,^19^,^20^,^21^,^22^. Root growth assays showed that *jazQ* seedlings have both an increased sensitivity to exogenous JA and a constitutive short-root phenotype in comparison to WT seedlings (Fig. 1b and Supplementary Fig. 1). We also compared the hormone sensitivity of *jazQ* seedlings to the *jaz10-1* mutant, which is one of the few *jaz* single mutants to exhibit enhanced sensitivity to JA^11^,^23^,^24^. Consistent with the notion that JAZ family members serve partially redundant roles in JA signalling, *jazQ* roots were significantly more sensitive to JA than the *jaz10-1* mutant (Supplementary Fig. 1). These findings support a key role for multiple JAZs in the control of root growth. Our data are also consistent with recent genetic analysis of the co-repressor Novel Interactor of JAZ (NINJA), which directly interacts with JAZs to negatively regulate JA signalling in roots^14^,^25^.

Analysis of defense-related phenotypes showed that glucosinolates and anthocyanins, whose biosynthesis in *Arabidopsis* is positively regulated by JA^22^,^26^, accumulate to higher levels in *jazQ* seedlings than WT (Fig. 1c,d). We also found that soil-grown *jazQ* plants had remarkably heightened resistance to attack by the generalist herbivore *Trichoplusia ni* (Fig. 1e). In contrast to these elevated defense traits, leaf area, petiole length and rosette dry weight were all reduced in *jazQ* relative to WT (Fig. 1f and Supplementary Fig. 2). *jazQ* also delayed the time to bolting but did not affect the number of leaves at the time of bolting (Supplementary Fig. 2). These results demonstrate that *jazQ* plants exhibit constitutive growth-defense antagonism (that is, reduced growth with enhanced defense) and thus provide a unique genetic model with which to interrogate how the JA branch of immunity is linked to growth.

### Loss of PHYB uncouples growth-defense tradeoffs in *jazQ*

**Figure 2:**
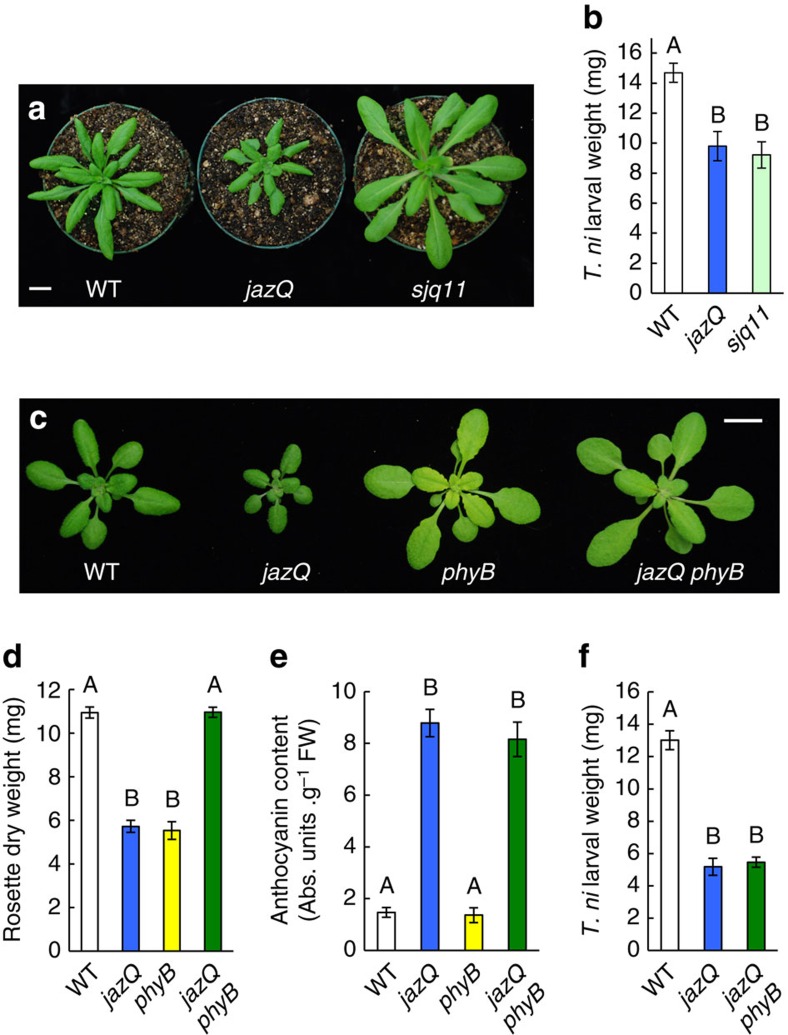
The combination of *jazQ* and *phyB* promotes robust growth of well-defended leaves. (**a**) Photograph of five-week-old WT, *jazQ* and *sjq11* plants, the latter of which is a suppressor mutant of *jazQ* harbouring a null mutation in *PHYB*. (**b**) *Trichoplusia ni* weight after feeding for 10 day on WT (31 larvae), *jazQ* (31 larvae) and *sjq11* (37 larvae) plants. Data show the mean±s.e.m. of at least 12 independent replicates. (**c**) Photograph of four-week-old plants grown in soil. (**d**,**e**) Rosette dry weight and anthocyanin accumulation in petioles, respectively. Data show the mean±s.e.m. of 10 plants per genotype. (**f**) *T. ni* weight after feeding for 10 days on WT (23 larvae), *jazQ* (29 larvae) and *jazQ phyB* (27 larvae) plants. Data show the mean larval weight±s.e.m. of insects reared on 12 plants per host genotype. Capital letters denote statistical differences according to Tukey HSD-test (*P*<0.05). Scale bars, 1 cm.

We visually screened an ethyl methanesulfonate (EMS) -mutagenized population of *jazQ* for mutants with increased rosette size and persistence of elevated leaf anthocyanin content. Among several *suppressor of jazQ* (*sjq*) mutants identified, one line (*sjq11*) showed a particularly striking leaf growth pattern that was heritable in subsequent generations (Fig. 2a). Importantly, bioassays performed with *T. ni* larvae showed that *sjq11* plants also maintained heightened resistance to herbivory (Fig. 2b). Initial characterization of *sjq11* revealed phenotypes similar to those described for phytochrome B (*phyB*) photoreceptor mutants, including early flowering time and elongated hypocotyls and petioles under continuous white light^27^. Genetic allelism tests and DNA sequencing confirmed that *sjq11* harbours a null mutation in the *PHYB* gene (Supplementary Fig. 3). To eliminate the possibility that additional EMS mutations contribute to the *sjq11* phenotype, further studies were performed with a *jazQ phyB* sextuple mutant obtained by crossing the reference *phyB-9* null allele into the *jazQ* background.

Analysis of growth and defense traits in *jazQ phyB* plants showed that the *jazQ* and *phyB* ‘single' mutant phenotypes were largely additive and often organ specific. For example, *jazQ phyB* roots retained the JA-hypersensitive phenotype of *jazQ*, whereas *jazQ phyB* hypocotyls showed the red-light insensitive phenotype of *phyB* (Supplementary Fig. 4). Adult *jazQ phyB* plants grown in soil resembled *phyB* in having elongated petioles, flattened rosette leaves^28^ and early flowering time (Fig. 2c and Supplementary Fig. 5). The *phyB* mutation is thus epistatic to *jazQ* for these growth traits. Interestingly, the rosette diameter, projected leaf area and dry mass of *jazQ phyB* rosette leaves under our growth conditions exceeded that of the *jazQ* and *phyB* parents, suggesting that the combination of *jazQ* and *phyB* has transgressive effects on leaf growth (Fig. 2d and Supplementary Fig. 5). Despite its robust vegetative growth, *jazQ phyB* plants maintained the heightened anti-insect defense and anthocyanin content of *jazQ* (Fig. 2e,f). The effect of combining *jazQ* and *phyB* on resistance to *T. ni* feeding was particularly striking because *phyB* alone causes high susceptibility to this herbivore (Supplementary Fig. 6). *jazQ* is thus epistatic to *phyB* with respect to leaf defense traits. These collective data demonstrate that *phyB* fully suppresses the diminutive growth stature of *jazQ* aerial organs but, remarkably, does not compromise the heightened anti-insect resistance that is imparted by *jazQ*.

### Co-expression of MYC and PIF regulons in *jazQ phyB*

A potential mechanistic explanation for the enhanced growth and defense attributes of *jazQ phyB* plants comes from recent studies that implicate crosstalk between the JA, phyB and GA signalling pathways in the modulation of growth-defense balance. Within this signalling network, GA stimulates cell extension growth by promoting the degradation of DELLA proteins that repress PIF transcription factors^29^ (Fig. 1a). Reciprocal antagonism between the JA and GA pathways involves JAZ-DELLA interactions that prevent these repressors from inhibiting their cognate transcription factors^19^,^20^,^30^. JA-GA crosstalk is integrated with the shade avoidance response through phyB-mediated perception of changes in the ratio of red to far red (R:FR) light. Low R:FR ratios indicative of leaf shading reduce phyB activity to relieve repression on PIFs, thereby promoting rapid growth through the concerted action of growth hormones such as auxin and brassinosteroid (Fig. 1a)^31^,^32^,^33^. Concurrent with this growth response to plant competitors, inactivation of phyB by low R:FR (or *phyB* mutation) is associated with depletion of DELLA proteins, increased JAZ stability, accelerated turnover of MYC transcription factors, and suppression of JA-triggered immune responses^5^,^9^,^34^.

These considerations led us to test the hypothesis that the combination of *jazQ* and *phyB* causes concomitant derepression of the MYC and PIF transcriptional programs to promote growth and defense simultaneously. In support of this idea, we found that overexpression of PIF4 in the *jazQ* background partially rescued the small rosette size and short petiole length of *jazQ* without affecting anthocyanin accumulation and resistance to *T. ni* feeding (Supplementary Fig. 7). This finding indicates that increased PIF4-mediated growth does not attenuate the defense stature of *jazQ* plants. The inability of PIF4 overexpression to fully recapitulate the *jazQ phyB* phenotype indicates that additional regulatory factors contribute to the growth vigour of *jazQ phyB*.

**Figure 3:**
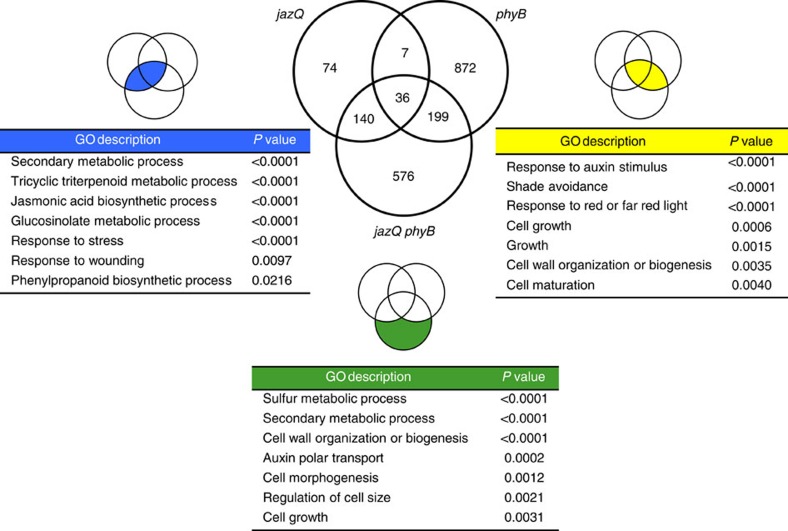
*jazQ* and *phyB* interact to promote expression of both growth and defense genes. WT, *jazQ*, *phyB* and *jazQ phyB* seedlings were grown for 8 days in continuous white light before RNA extraction and analysis of gene expression by mRNA sequencing. The Venn diagram shows the number of genes upregulated in comparisons between WT and each of the three mutants. GO analysis of functional categories was performed with gene sets that are shared between *jazQ* and *jazQ phyB* (blue intersect), shared between *phyB* and *jazQ phyB* (yellow intersect), or unique to *jazQ phyB* (green shade). See Supplementary Data 1 for detailed expression data and GO analysis.

To further test the hypothesis that MYC and PIF transcriptional modules are simultaneously activated in *jazQ phyB* plants, we used mRNA sequencing to compare the transcript profile of WT, *jazQ*, *phyB*, and *jazQ phyB* seedlings. ‘Secondary metabolism' and ‘response to stress' and were among the biological processes most significantly overrepresented in ontologies of 257 genes expressed to higher levels in *jazQ* than WT (Fig. 3 and Supplementary Data 1). This gene set included glucosinolate biosynthesis genes that are direct targets of MYC2 (ref. 22) (Supplementary Fig. 8), as well as genes involved in the synthesis of triterpenoids, jasmonates, and various defensive proteins (Supplementary Fig. 9). In agreement with their enhanced defense stature, *jazQ phyB* plants maintained increased expression of the majority (68%) of genes that are upregulated in *jazQ* (Fig. 3, Supplementary Figs 8 and 9). Analysis of growth-related genes revealed that the set of 235 genes upregulated in both *phyB* and *jazQ phyB* is enriched for functional classes involved in responses to auxin, shade avoidance, cell wall organization and light stimulus (Fig. 3). Several genes within this group were previously shown to be direct targets for PIF transcription factor binding^33^,^35^,^36^ (Supplementary Fig. 9). These data provide evidence that the combination of *jazQ* and *phyB* promotes concomitant expression of defense and growth-related genes, at least in part, via the concurrent activation of MYC and PIF transcriptional modules.

Among the 576 transcripts whose abundance was significantly increased in *jazQ phyB* but not *jazQ* or *phyB*, there was a strong over-representation of GO terms related to secondary metabolism, cell wall organization, growth and auxin transport (Fig. 3). These data suggest that the combination of *jazQ* and *phyB* has synergistic effects on the expression of certain growth and defense responses in *jazQ phyB*. In support of this idea, quantitative PCR analysis showed that wound-induced expression of select JA-response genes was significantly higher in *jazQ phyB* than WT leaves, which may contribute to the heightened defense of *jazQ phyB* plants relative to WT (Supplementary Fig. 10). It is possible that these synergistic effects of *jazQ* and *phyB* on gene expression result from functional interaction between MYCs and PIFs at the level of protein–protein interaction or altered binding to common *cis*-regulatory elements in target genes^21^,^33^,^34^,^37^. Supporting this hypothesis, PIF4 targets previously identified by ChIP-seq^33^ include an over-representation of genes associated with the terms ‘jasmonate stimulus' and ‘response to wounding' (Supplementary Table 1). Moreover, several PIF4 targets are also targets of MYC2 (Supplementary Table 2). We cannot exclude the possibility that concurrent loss of phyB and JAZ1/3/4/9/10 affects the activity of transcriptional regulators other than PIFs and MYCs to contribute to the gene expression profile and other general phenotypes of *jazQ phyB* plants.

### Genetic modulation of photosynthesis and leaf architecture

**Figure 4:**
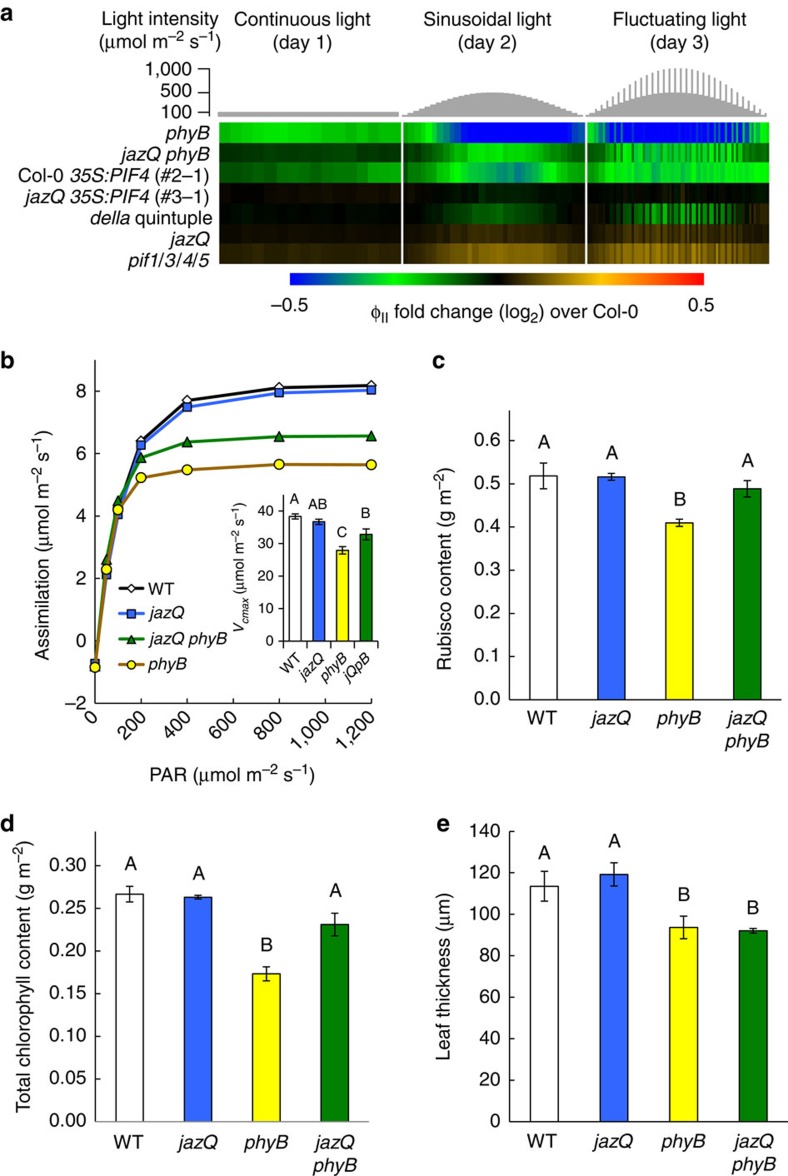
*jazQ* and *phyB* interact to modulate photosynthesis and leaf architecture. (**a**) Heat map of photosystem II quantum efficiency (Φ_II_) in response to varying light regimes. Chlorophyll fluorescence values for the indicated mutants were normalized to Col-0. Plants were exposed to three consecutive 16 h day^−1^ light regimes: constant light (day 1, left); sinusoidal increase and decrease in light intensity (day 2, middle); and sinusoidal light with higher intensity pulses (day 3, right). Statistical analysis of data is shown in Supplementary Fig. 12. (**b**) Photosynthetic rate in response to increasing light measured by gas exchange in 6–9 plants per genotype. Inset shows nonlinear curve-fitting to model the maximum velocity of Rubisco determined from foliage photosynthetic rates in response to increasing CO_2_. (**c**) Rubisco and (**d**) total chlorophyll concentration in leaves from 54-days-old plants (*n*=4). (**e**) Thickness of 22-day-old rosette leaves (*n*=4). In **b**–**e**, data show the mean±s.e.m., and capital letters indicate statistical difference at *P*<0.05 (Tukey HSD-test). In **d**, WT and *jazQ phyB* means are different at *P*<0.1.

The robust growth of well-defended *jazQ phyB* leaves led us to investigate whether *jazQ* and *phyB* interact to modulate leaf photosynthetic efficiency. Indeed, PIF activity is known to repress chloroplast development and photosynthetic competency^37^, and we observed that ‘photosynthesis' was the term most significantly overrepresented among genes that are repressed in both *phyB* and *jazQ phyB* seedlings (Supplementary Fig. 11 and Supplementary Data 1). We employed non-invasive, whole-plant chlorophyll fluorescence imaging^13^,^38^ to determine how genetic perturbations within the phyB-GA-JA signalling network affect photosystem II efficiency (Φ_II_) under various light regimes, including those designed to simulate natural environments (Fig. 4a and Supplementary Fig. 12). *phyB* plants had reduced Φ_II_ under continuous low light intensity and this effect was exacerbated under the sinusoidal and fluctuating light regimes, consistent with previous studies^39^. A decrease in Φ_II_ was also observed in a Col-0 transgenic line (*35S:PIF4*) that overexpresses PIF4. Interestingly, the negative effect of *phyB* and *35S:PIF4* on photosynthetic efficiency was rescued by *jazQ*, which alone had little (or very weak positive) effect on Φ_II_ (Fig. 4a and Supplementary Fig. 12). Consistent with the role of PIFs in repressing photosynthesis, a *pif1/3/4/5* quadruple mutant (*pifq*) showed increased Φ_II_ under fluctuating light conditions, whereas loss of DELLAs in the *della* quintuple mutant (*dellaQ*) reduced Φ_II_ under these conditions. That photosystem II efficiency was lower in *phyB* than in *dellaQ* leaves suggests that phyB exerts stronger repression on PIF activity than DELLA proteins under these growth conditions.

To obtain additional insight into physiological processes that underlie growth-defense vigour of *jazQ phyB*, we investigated the relationship between photosynthesis and leaf growth to obtain an estimate of leaf construction costs^40^. Gas exchange experiments showed that *phyB* leaves have significantly lower photosynthetic rate per unit leaf area, whereas photosynthetic capacity of *jazQ* on a leaf area or dry weight basis was comparable to WT (Fig. 4b), consistent with our chlorophyll fluorescence measurements. *phyB* leaves also contained less area-based chlorophyll and Rubisco (D-ribulose-1,5-bisphosphate carboxylase/oxygenase) than WT. Modelling of photosynthetic parameters showed that the reduced photosynthetic capacity of *phyB* at high light results in part from a limitation in Rubisco activity. *jazQ* partially rescued the low photosynthetic capacity of *phyB* leaves, as well as the low area-based Rubisco and chlorophyll content of *phyB* (Fig. 4c,d). The mechanism by which *jazQ* partially restores the reduced photosynthetic performance of *phyB* leaves in unknown but deserves further attention.

We also found that *phyB* leaves were thinner than those of WT and *jazQ* and that this trait was retained in *jazQ phyB* (Fig. 4e). Because of the greater projected leaf area available to intercept light (due to longer petioles and flatter, thinner leaves), the whole-plant photosynthetic rate in *jazQ phyB* plants was similar to WT. Thus, costs associated with construction of *jazQ phyB* leaves may be lowered through increased partitioning of carbon to leaf area at the expense of leaf thickness^40^. These data suggest that changes in leaf architecture rather than increased efficiency of the photosynthetic apparatus may contribute to the growth-defense vigour of *jazQ phyB* relative to WT plants.

## Discussion

A key innovation of our study was the development of a JA signalling mutant (*jazQ*), in which the removal of multiple JAZ repressors causes hyperactivation of JA responses. As a consequence, *jazQ* plants exhibit both enhanced resistance to insect herbivory and diminished growth of leaves and roots. The heightened defense of *jazQ* plants most likely involves increased activity of MYC transcription factors, which are well characterized for their role in promoting secondary metabolism and defense^21^,^22^. Our results also provide evidence that transcriptional changes account for the growth phenotypes of *jazQ*, but the molecular basis of growth restraint remains to be determined. DELLA proteins have previously been implicated in JA-mediated growth inhibition of *Arabidopsis* roots^19^ and hypocotyls^20^. Although these studies raise the possibility that removal of JAZs in *jazQ* increases the growth-inhibiting activity of DELLAs, previous studies showed that DELLA proteins are not required for wound- and JA-induced growth stunting of leaves^12^. Given the high connectivity of JAZs within protein interaction networks and their emerging role as integrators of various signalling pathways^41^,^42^, it is possible that JAZ-interacting transcriptional regulators other than MYCs and DELLAs also contribute to general phenotypes of *jazQ*. Further analysis of *jazQ* should provide new avenues to study how JA controls plant growth and development.

By exploiting *jazQ* phenotypes in a genetic suppressor screen, we identified *jazQ phyB* as a novel genotypic combination that uncouples growth-defense tradeoffs mediated by the JA signalling pathway. This major conclusion is based on the finding that *phyB* suppresses the growth restriction but not enhanced defense of *jazQ* leaves. Previous studies of phyB-JA crosstalk showed that decreased levels of the active form (Pfr) of phyB, as a consequence of either *phyB* mutation or exposure to shade light, compromises JA-mediated resistance to a broad spectrum of biotic attackers^5^,^31^,^32^. Recent molecular evidence further indicates that shade-triggered repression of JA-mediated defense in *Arabidopsis* involves stabilization of JAZs and reduced MYC activity^9^,^34^. Given these findings, a plausible mechanistic explanation for why *jazQ*-mediated leaf defense remains elevated in the presence of *phyB* is that one or more of the mutated *JAZ* genes in *jazQ* (*JAZ1/3/4/9/10*) are critical for suppressing JA-mediated defenses when levels of active phyB are reduced (for example, in the *phyB* genetic background). Consistent with this, analysis of the *jaz10-1* single mutant has shown that *JAZ10* is required for attenuation of JA-mediated defense by shade light^9^,^43^,^44^. Future studies are needed to ascribe specific functions to individual *JAZ* family members and, more generally, to better understand how light and defense perception pathways are integrated to achieve growth-balance in specific tissues.

A central premise underlying current views of growth-defense balance is that the production of defensive compounds and associated morphological structures is energetically costly. The ability of *jazQ phyB* leaves to grow and defend well at the same time provides evidence that JA-mediated growth-defense antagonism is not simply a consequence of ‘metabolic competition' that shunts resources to defense at the expense of growth^6^,^7^,^45^. Our data support an alternative (but not mutually exclusive) model, in which tradeoffs between growth and defense are orchestrated by a hormone-based transcriptional network that is hardwired to restrict growth upon activation of the JA signalling pathway. This interpretation is bolstered by an increasing number of molecular studies that have addressed to role of hormones in the intersection of growth and immunity^4^,^20^,^46^,^47^. In light of current theories regarding the costs of induced defense^45^, it remains to be determined whether growth inhibition resulting from genetic removal (for example, *jazQ*) or natural JA-induced degradation of JAZs provides a fitness benefit to the plant. It is conceivable, for example, that JA-mediated growth restriction enhances the plant's capacity to anticipate future threats or to cope with multiple-stress conditions^48^. This hypothesis predicts that simultaneous loss of phyB and JAZ, as major drivers of phenotypic plasticity, may reduce the fitness of *jazQ phyB* plants in natural environments because these plants lack the regulatory potential to adapt to complex and rapidly changing environments. Support for this idea comes from our RNA-seq data showing that the expression of genes involved in abiotic stress response is repressed in *jazQ phyB* (Supplementary Fig. 11). An important future direction will thus be to assess the reproductive fitness of these genotypes in the presence of various predators, competitors and abiotic stressors.

The ability to unlock growth-defense tradeoffs through relief of transcription repression may provide an approach to assemble plant traits in ways that have practical application in biotechnology and agronomy. One example concerns the use of exogenous JA to elicit the biosynthesis of commercially valuable plant secondary metabolites^49^,^50^. A significant bottleneck in JA-elicited production of these compounds, including the anti-cancer drug paclitaxel (taxol), is the loss of plant biomass resulting from JA-mediated growth inhibition^50^,^51^. The design of plants with increased flux through JA-responsive metabolic pathways without concomitant growth restriction could provide a solution to this problem. Similarly, unlinking growth-defense antagonism may be useful in cropping systems that increasingly depend upon high planting densities to maximize yield. Because the ratio of R:FR light perceived by canopy leaves decreases as planting densities increase, densely planted crops may become more susceptible insect pests and pathogens that are sensitive to JA-mediated defenses^31^,^32^. In demonstrating that *jazQ* and *phyB* can be combined to produce what is essentially a well-defended shade leaf, our results suggest a strategy to cultivate densely planted crops with less dependence on pesticides. The evolutionary conservation of light and defense signalling pathways suggest that the findings described here can readily be applied to most plant species using modern genetic approaches.

## Methods

### Plant material and growth conditions

*A. thaliana* Columbia ecotype (Col-0) was used as a WT parent for all experiments. Soil-grown plants were maintained at 20 °C (±1 °C) under 16 h at a light intensity of 120 μE m^−2^ s^−1^ and 8 h dark unless otherwise noted. For the first 10 day after seed sowing, trays containing potted plants were covered with a transparent plastic dome to increase humidity. For experiments involving growth of seedlings on agar plates, seeds were surface sterilized for 15 min in a solution containing 50% (v/v) bleach and 0.1% (v/v) Triton X-100, washed 10 times with sterile water and then stratified in dark at 4 °C for 2 days. Seeds were then sown on 0.7% (w/v) agar media containing half-strength Murashige and Skoog (MS; Caisson Labs) salts supplemented with 0.8% (w/v) sucrose. Transfer DNA (T-DNA) insertion mutants used for construction of *jazQ* were obtained from the *Arabidopsis* Biological Research Center (ABRC; The Ohio State University) and named as follows: *jaz1-2* (JIC-SM.22668), *jaz3-4* (GK-097F09), *jaz4-1* (SALK_141628), *jaz9-4* (GK-265H05) and *jaz10-1* (SAIL_92_D08). *jaz3-4* and *jaz9-4* lines were backcrossed to Col-0 to remove unlinked T-DNA insertions. *jaz10-1* was backcrossed to Col-0 to remove a *qrt1-2* mutation present in the SAIL lines^52^. *jaz4-1* and *jaz10-1* mutants have been described^23^,^53^. The *jazQ phyB* sextuple mutant was obtained from a genetic cross between *jazQ* and the *phyB* reference allele *phyB-9* (ref. 27). The higher-order *pifq* (*pif1-1/pif3-3/pif4-2/pif5-3*) and *dellaQ* (*gai-t6/rgat2/rgl1-1/rgl2-1/rgl3-1*) mutants have been described^54^,^55^.

### PCR analysis

PCR-based genotyping of *jazQ* and lower-order mutants relied on primer sets flanking T-DNA insertion sites, together with a third primer recognizing the border of the inserted T-DNA. The forward, reverse, and border primers used were the following: *JAZ1* (At1g19180), 5′-ACCGAGACACATTCCCGATT-3′, 5′-CATCAGGCTTGCATGCCATT-3′, and 5′-ACGAATAAGAGCGTCCATTTTAGAG-3′; *JAZ3* (At3g17860), 5′-ACGGTTCCTCTATGCCTCAAGTC-3′, 5′-GTGGAGTGGTCTAAAGCAACCTTC-3′, and 5′-ATAACGCTGCGGACATCTACATT-3′; *JAZ4* (At1g48500), 5′-TCAGGAAGACAGAGTGTTCCC-3′, 5′-TGCGTTTCTCTAAGAACCGAG-3′, and 5′-TTGGGTGATGGTTCACGTAG-3′; *JAZ9* (At1g70700), 5′-TACCGCATAATCATGGTCGTC-3′, 5′-TCATGCTCATTGCATTAGTCG-3′, and 5′-CTTTGAAGACGTGGTTGGAACG-3′; *JAZ10* (At5g13220), 5′-ATTTCTCGATCGCCGTCGTAGT-3′, 5′-GCCAAAGAGCTTTGGTCTTAGAGTG-3′, and 5′-GTCTAAGCGTCAATTTGTTTACACC-3′. PCR with reverse transcription (RT–PCR) was used to confirm the presence or absence of *JAZ* transcripts in WT and *jazQ* plants. For this purpose, RNA was extracted from 8-day-old seedlings grown on MS plates containing 20 μM MeJA. Frozen tissue was homogenized with a mortar and pestle and RNA was extracted using an RNeasy kit (Qiagen) with on-column DNase (Qiagen) treatment. cDNA was reverse transcribed from 1 μg total RNA with a High-Capacity cDNA Reverse Transcription kit (Applied Biosystems, ABI). RT–PCR was performed using primer sets designed to amplify the five *JAZ* genes and the internal control *ACTIN1* (At2g37620). The forward and reverse primer sets used were as follows: *JAZ1*, 5′-ATGTCGAGTTCTATGGAATGTTCTG-3′ and 5′-TCATATTTCAGCTGCTAAACCGAGCC-3′; *JAZ3*, 5′-ATGGAGAGAGATTTTCTCGGG-3′ and 5′-TTAGGTTGCAGAGCTGAGAGAAG-3′; *JAZ4*, 5′-ATGGAGAGAGATTTTCTCGG-3′ and 5′-CAGATGATGAGCTGGAGGAC-3′; *JAZ9*, 5′-ATGGAAAGAGATTTTCTGGGTTTG-3′ and 5′-TTATGTAGGAGAAGTAGAAGAGTAATTCA-3′; *JAZ10*, 5′-ATGTCGAAAGCTACCATAGAAC-3′ and 5′-GATAGTAAGGAGATGTTGATACTAATCTCT-3′; and *ACTIN1*, 5′-ATGGCTGATGGTGAAGACATTCAA-3′ and 5′-TCAGAAGCACTTCCTGTGAACAAT-3′. RT–PCR reactions were performed with the following conditions: 94 °C for 5 min, followed by 30 cycles of denaturation (45 s at 94 °C), annealing (30 s at 52 °C) and elongation (1.5 min at 72 °C). Final elongation step was performed at 72 °C for 10 min and completed reactions were maintained at 12 °C. Forty elongation cycles were used to detect the *JAZ4* transcripts, which accumulate at low levels in WT plants^56^.

### Root growth assays

The effect of exogenous JA on seedling root growth inhibition was determined by growing seedlings on square Petri plates (Fisher) containing MS medium supplemented with the indicated concentration of methyl-JA (MeJA; Sigma-Aldrich)^57^. Plates were incubated vertically in a growth chamber maintained at 21 °C under continuous light for 8 days. Primary root length was measured using the ImageJ software (http://imagej.nih.gov/ij/). WT and mutant lines were grown on the same plate to control for plate-to-plate variation.

### Quantification of secondary metabolites

Anthocyanins were quantified using established procedures^58^, with the following modifications. Petioles were excised from 4-week-old plants and extracted in 1 ml MeOH containing 1% (v/v) HCl. Samples were incubated overnight at 4 °C with constant agitation. Anthocyanin pigments in the resulting extract were measured spectrophotometrically and calculated as A_530_−0.25(A_657_) g^−1^ fresh weight. Glucosinolates were quantified using 8-day-old seedlings grown on solid MS medium^59^. Seedlings were collected into 2 ml tubes (∼50 seedlings per tube) and immediately frozen in liquid nitrogen. WT and mutant lines were grown on the same plate to avoid plate-to-plate variation. Frozen tissue was lyophilized, ground to a fine powder and extracted with 1 ml 80% MeOH containing an internal standard (25 nmol sinigrin, Sigma-Aldrich). Samples were briefly vortexed, incubated at 75 °C for 15 min, and then centrifuged at 23 °C at 10,000*g* for 10 min. Resulting supernatants were applied to Sephadex A-25 columns (Amersham). Glucosinolates were released from the columns as desulfoglucosinolates with a solution containing 30 μl of aryl sulfatase (3.0 mg ml^−1^; Sigma) and 70 μl water (high-performance liquid chromatography grade). Following an overnight incubation in the dark at 23 °C, samples were eluted from the columns with 200 μl 80% MeOH and 200 μl water. Samples were then lyophilized to complete dryness and re-dissolved in 100 μl water. Desulfoglucosinoaltes were detected by high-performance liquid chromatography^59^. Compound abbreviations in Fig. 1c correspond to the following: 3MSP, 3-methylsulfinylpropylglucosinolate; 4MSB, 4-methylsulfinylbutylglucosinolate; 7MSH, 7-methylsulfinylheptylglucosinolate; 4MTB, 4-methylthiobutylglucosinolate; 8MSO, 8-methylsulfinyloctylglucosinolate; I3M, indol-3-ylmethylglucosinolate; 4MI3M, 4-methoxyindol-3-ylmethylglucosinolate; 1MI3M, 1-methoxyindol-3-ylmethylglucosinolate and 8MTO, 8-methylthiooctylglucosinolate.

### Insect-feeding assays

Insect-feeding assays were performed with soil-grown plants maintained in a growth chamber at 19 °C and a photoperiod of 8 h light (120 μE m^−2^ s^−1^) and 16 h dark. Neonate *Trichoplusia ni* larvae (Benzon Research) were transferred to the centre of fully expanded rosette leaves of 6-week-old plants^60^. Four larvae were reared on each of 12 plants per genotype. Plants were then covered with a transparent dome and returned to the chamber for 10 days, after which larval weights were measured.

### Growth and flowering time measurements

Three-to-four week-old soil-grown plants were used for all measurements (typically 10 plants per measurement), unless indicated otherwise. Petiole length of the third true leaf was measured with a caliper after leaf excision. Bolting time was measured in a separate set of plants by counting the number of true leaves on the main stem and the number of days from sowing until bolting (that is, floral buds visible in the centre of the rosette). The same set of plants was subsequently used to assess the length of time to opening of the first flower. Rosette diameter and leaf area were determined by photographing rosettes from the top with a Nikon D80 camera. The resulting images were used to calculate Feret diameter using ImageJ analysis. Total leaf area was determined with GIMP software (http://www.gimp.org). Leaf dry weight was determined by weighing excised rosettes (without roots) after freeze drying for 2 days in a lyophilizer.

### *jazQ* suppressor screen and identification of *sjq11*

Approximately 50,000 *jazQ* seeds were mutagenized by immersion in a solution of 0.1% or 0.2% (v/v) EMS (Sigma-Aldrich) for 16 h at room temperature, with constant agitation. Seeds (M_1_ generation) were thoroughly washed with H_2_O, stratified in the dark at 4 °C for 2 days and then immediately sown on soil. M_2_ seed was collected from 16 pools of self-pollinated M_1_ plants (approximately 1,000 M_1_ plants/pool). Soil-grown M_2_ plants (∼2,000 plants/pool) were visually screened for individuals having a larger rosette size than *jazQ*. Putative *sjq* (*suppressors of the jazQ*) mutants were rescreened in the M_3_ generation to confirm heritability of phenotypes. Insight into the causal mutation in *sjq11* came from the observation that *sjq11* seedlings grown on MS medium in continuous white light for 3 days have elongated hypocotyls. Subsequent hypocotyl growth assays in monochromatic red light^61^ confirmed a defect in red-light signalling. Briefly, *sjq11* (M_3_ generation) and control seeds were plated on MS medium lacking sucrose and stratified at 4 °C in dark for 2 days. Mutant and control lines were grown on the same plate to control for plate-to-plate variation. A 3 h pulse of white light was then administered to improve synchronous seed germination. Plates were then returned to darkness for one d at 21 °C and then transferred to a monochromatic light-emitting diode chamber outfitted to emit red light (670±20 nm; 25 μE μE m^−2^ s^−1^). As a control, a set of plates containing each genotype was maintained in darkness. Following 3 days of growth, seedling hypocotyls were measured by ImageJ software analysis of scanned images. Allelism tests performed with F_1_ seedlings (obtained from the cross between *sjq11* and *phyB-9*) revealed a lack of genetic complementation. Finally, sequencing of the *PHYB* gene (AT2G18790) in *sjq11* revealed a C→T transition that introduces a stop codon in a region of the gene that encodes the chromophore-binding domain of phyB.

### Gene expression profiling

Global gene expression profiling in 8-day-old whole seedlings (Col-0 WT, *jazQ*, *phyB-9*, *jazQ phyB-9*) was assessed by mRNA sequencing (RNA-seq) performed on the Ilumina HiSeq 2000 platform. Seedlings were grown in continuous light on solid MS medium supplemented with sucrose. For each replicate sample, ∼200 seedlings were harvested for RNA extraction. WT and mutant seedlings were grown on the same plate to minimize plate-to-plate variation. Three independent RNA samples (biological replicates) were sequenced per genotype. Total RNA was isolated as described above and RNA integrity was assessed with a 2100 Bioanalyzer (Agilent Technologies). All samples utilized had an integrity score of at least 7.0. Single-end (50 bp) sequencing was performed at the Michigan State University Research Technologies Service Facility (https://rtsf.natsci.msu.edu). Barcoded sequencing libraries were constructed using the Illumina RNAseq kit according to the manufacturer's instructions and were multiplexed in six libraries per lane. The average number of sequencing reads was 18.4±4.3 million per sample. Raw sequencing reads were assessed with Illumina quality control tools filters and FASTX toolkit (http://hannonlab.cshl.edu /fastx_toolkit/). Reads were mapped to gene models in TAIR10 with the program RSEM (version 1.2.11) set for default parameters^62^. Data was expressed as transcripts per million. The average transcripts per million±s.e.m. for all *Arabidopsis* genes is provided in Supplementary Data 1. DESeq (version 1.18.0) (ref. 63) was used to normalize expected counts from RSEM and to assess differential gene expression by comparing normalized counts in WT to those in a particular mutant. Gene ontology (GO) analysis of enriched functional categories was performed using BiNGO (version 2.44) (ref. 64). The hypergeometric test with Benjamini & Hochberg's FDR correction was used to calculate over- and underrepresented GO categories among differentially expressed genes, using a *P* value<0.05.

For wounding experiments, 3-week old soil-grown seedlings were wounded twice across the midvein of four leaves (leaves 3–6, counted from first rosette leaf). After 1 h, the wounded leaves of two plants were pooled and immediately frozen in liquid nitrogen. Equivalent leaves of two unwounded plants were pooled and collected as controls. The experiment was independently replicated twice, with each experiment consisting of 3–4 biological replicates. Frozen tissue was homogenized with a TissueLyser II (Qiagen) and RNA was extracted using an RNeasy kit (Qiagen) with on-column DNase (Qiagen) treatment, as per the manufacturer's instructions. RNA quality was assessed by *A*_*260*_/*A*_*280*_ ratios using a ND-1000 UV Nanodrop spectrophotometer (Thermo Scientific). cDNA was reverse transcribed using a High-Capacity cDNA Reverse Transcription kit (Applied Biosystems, ABI), as per the manufacturer's instructions, and cDNA was diluted to 0.5 ng μl^−1^ with nuclease-free water. Quantitative real-time PCR reactions consisted of 5 μl of 2 × Power SYBR Green (ABI) master mix, 2 ul diluted cDNA template (1 ng total), 1 μl 5 uM forward and reverse primers, and nuclease-free water for 10 μl total reaction volume. The forward and reverse primers used were the following: *PP2A*, 5′-AAGCAGCGTAATCGGTAGG-3′ and 5′-GCACAGCAATCGGGTATAAAG-3′; *AOS*, 5′-GGAGAACTCACGATGGGAGCGATT-3′ and 5′-GCGTCGTGGCTTTCGATAACCAGA-3′; *LOX3*, 5′-GCTGGCGGTTCGACATG-3′ and 5′-GCCATTCCTCTGCGAATTAGA-3′; and *MYC2*, 5′-AGAAACTCCAAATCAAGAACCAGCTC-3′ and 5′-CCGGTTTAATCGAAGAACACGAAGAC-3′. Reactions were run on an ABI 7500 Fast qPCR instrument with the following conditions: 95 °C for 10 min, then 40 cycles of 15 s at 95 °C for denaturation and 60 s at 60 °C for annealing and polymerization. A dissociation curve was performed at the end of each reaction to confirm primer specificity using default parameters (15 s at 95 °C, 60 s at 60 °C–95 °C in 1 °C increments, and 15 s at 95 °C). Target gene expression was normalized to the expression of *PP2a*, which is stable under JA-inducing conditions^13^. The normalization incorporated primer efficiencies determined for each primer pair using LinRegPCR v2012.0 (ref. 65) from the log-linear phase of each amplification plot.

### Overexpression of *PIF4* in the *jazQ* background

The *35S::PIF4*-TAP overexpression construct^66^ was kindly provided by Dr Michael Thomashow (Michigan State University). Transformation of *jazQ* plants with *Agrobacterium tumefaciens* (strain C58C1) was performed using the flower dip method^67^. Multiple independent transformed lines (T1 generation) were selected on MS plates containing gentamycin and transferred to soil for subsequent analysis. Homozygous lines were selected by testing the T3 progeny for gentamycin resistance.

### Photosynthesis measurements

Gas exchange measurements were performed with plants grown in plastic containers (‘Cone-tainers', Steuwe and Sons, Tangent, OR, USA) on an 8 h light (19 °C)/ 16 h dark (16 °C) photoperiod and 120 μmol m^−2^ s^−1^ photosynthetic photon flux density (PPFD)^40^,^68^. Single mature rosette leaves (attached) from 8- to 10-week-old plants were used to obtain CO_2_ response curves on a LI-6400XT system (LI-COR Biosciences, Lincoln, NE, USA) outfitted with a standard leaf chamber (chamber area=6 cm^2^). Leaves were supplied with an artificial air mixture consisting of 20% O_2_, 80% N_2_, and 400 ppm CO_2_ at intensity of light 500 μmol m^−2^ s^−1^. Leaf temperature was maintained at ∼20 °C (block temperature set to 18 °C). Leaves were acclimated under this condition for at least 30 min before the start of each experiment. Assimilation rates were normalized to projected leaf area as measured by image analysis with the GIMP software. Area- and whole-plant-based photosynthesis and respiration was determined at four time points of the *Arabidopsis* growth cycle using plants grown under short-day conditions^40^.

*In situ* chlorophyll fluorescence measurements were performed in a Percival AR41L2 (Geneva Scientific, http://www.geneva-scientific.com) refitted as a Dynamic Environment Photosynthesis Imager (DEPI) system^13^,^38^. Images were processed using visual phenomics software^69^. The quantum yield of PSII (ΦII) was calculated as (*F*'_M_−*F*_S_)/*F*'_M_, where *F*_S_ is the steady-state fluorescence and *F*'_M_ is the fluorescence maximum at steady state. Statistical analysis of the ΦII data is provided in Supplementary Fig. 12.

### Leaf thickness measurements

Leaf cross sections obtained from the 5th leaf of 22-day old rosette leaves were examined under an Olympus FluoView FV1000, Confocal Laser Scanning Microscope (Olympus, NJ, USA) in the Center for Advanced Microscopy, Michigan State University. Leaf thickness was measured as the distance between the abaxial and adaxial surfaces of the leaf^40^.

### Measurement of total chlorophyll and Rubisco

Chlorophyll was extracted from 54-d old *Arabidopsis* rosette leaves with 96% ethanol. Absorbance of the extracted chlorophyll was measured spectrophotometrically at 665 nm and 649 nm and the total chlorophyll was calculated using the following equation: Chl_a_+Chl_b_=(13.95*A*_665_–6.88*A*_649_)+(24.96*A*_649_–7.32*A*_665_)^70^. Total protein was extracted from 54-d old *Arabidopsis* rosette leaves using a Plant Total Protein Extraction Kit (Sigma-Aldrich, MO, USA). A modified Lowry Assay was performed to measure the total protein concentration in the extract and the purity and quality of the extracted protein were determined by denaturing polyacrylamide gel electrophoresis. Equal amounts of total protein were loaded onto an automated capillary-based size western blotting system (ProteinSimple Wes System, San Jose CA, USA). All procedures were performed with manufacturer's reagents according to their user manual. Protein separation and immunodetection were performed automatically on the individual capillaries using the default settings. Antibodies raised against the large subunit of Rubisco (rabbit, AS03 037; Agrisera, Sweden; dilution used 1:650) were used to detect Rubisco in each protein sample. For quantification, all subsequent data generated was analysed with Compass Software provided by manufacturer (ProteinSimple, San Jose CA). Peak heights of the fluorescence signals were used to calculate relative differences of Rubisco concentration between samples. Rubisco concentration per unit leaf area was calculated based on the total protein concentration and measurements of leaf area per unit mass.

### Data Availability

RNA sequencing data is deposited at the National Center for Biotechnology Information Gene Expression Omnibus (GEO) as series record GSE79012. The authors declare that all other data supporting the findings of this study are available within the article and its Supplementary Information files or are available from the corresponding author upon request.

## Additional information

**How to cite this article**: Campos, M. L. *et al*. Rewiring of jasmonate and phytochrome B signalling uncouples plant growth-defense tradeoffs. *Nat. Commun.* 7:12570 doi: 10.1038/ncomms12570 (2016).

## Supplementary Material

Supplementary Information Supplementary Figures 1-12, Supplementary Tables 1-2 and Supplementary References

Supplementary Data 1 Full dataset comprising RNAseq and gene onthology (GO) analysis performed on WT, *jazQ*, *phyB* and *jazQ phyB* seedlings

Peer Review File
