# Supplementary material for: Rewiring of jasmonate and phytochrome B signalling uncouples plant growth-defense tradeoffs
Source: Nat Commun. 2016 Aug 30;7:12570. doi: 10.1038/ncomms12570 (PMC5155487; doi:10.1038/ncomms12570)
Supplement: Supplementary Information — Supplementary Figures 1-12, Supplementary Tables 1-2 and Supplementary References [file ncomms12570-s1.pdf]

Supplementary Information for Campos *et al*.

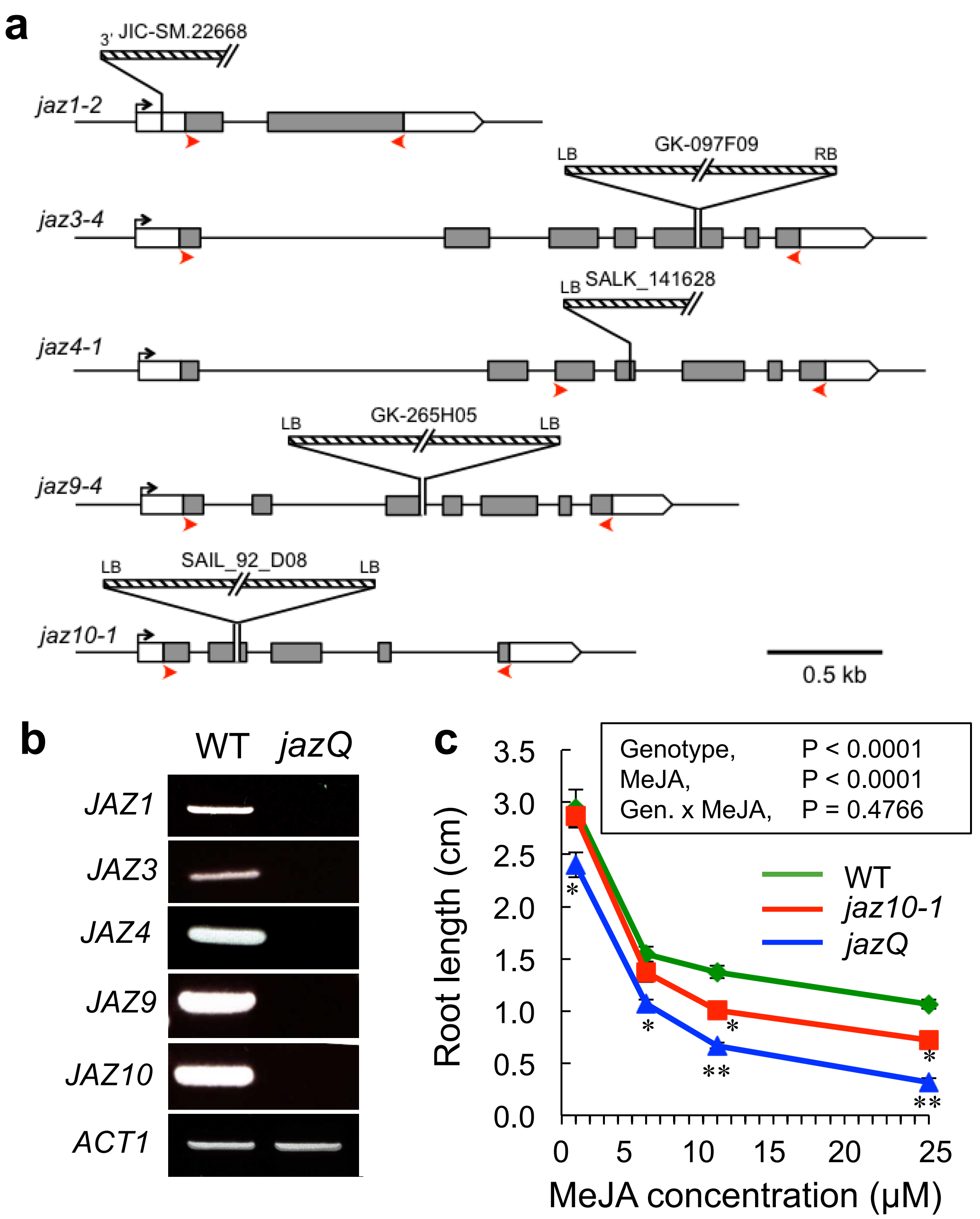

Supplementary Figure 1. A *jaz* quintuple (*jazQ*) mutant is highly sensitive to JA. a, Schematic of T-DNA insertion lines used for construction of *jazQ*. The organization of each *JAZ* gene is depicted by white and grey boxes representing untranslated regions (UTRs) and exons, respectively. The identity and position of the T-DNA insertion is shown. Red arrows show the position of primers used to test expression by RT-PCR. **b**, RT-PCR analysis of *JAZ* gene expression in WT and *jazQ*. RNA was obtained from seedlings grown for eight d on plates containing 25 μM MeJA. The *ACTIN1* gene (*ACT1,* At2g37620) was used as a positive control. **c**, Root length of WT, *jaz10-1*, and *jazQ* mutant seedlings grown for eight d on MS medium supplemented with 5, 10 or 25 μM MeJA. Control seedlings were grown in the absence of MeJA (0 μM). Data show the mean ± s.e. of measurements on at least 12 seedlings per genotype. P-values are shown for two-way ANOVA comparisons (inset). Asterisks represent statistical difference between mutant and WT according to Tukey HSD test (P < 0.05). Single asterisks denote a significant difference between mutant and WT, whereas double asterisks denote a significant difference between *jaz10-1* and *jazQ* at a given concentration of MeJA.

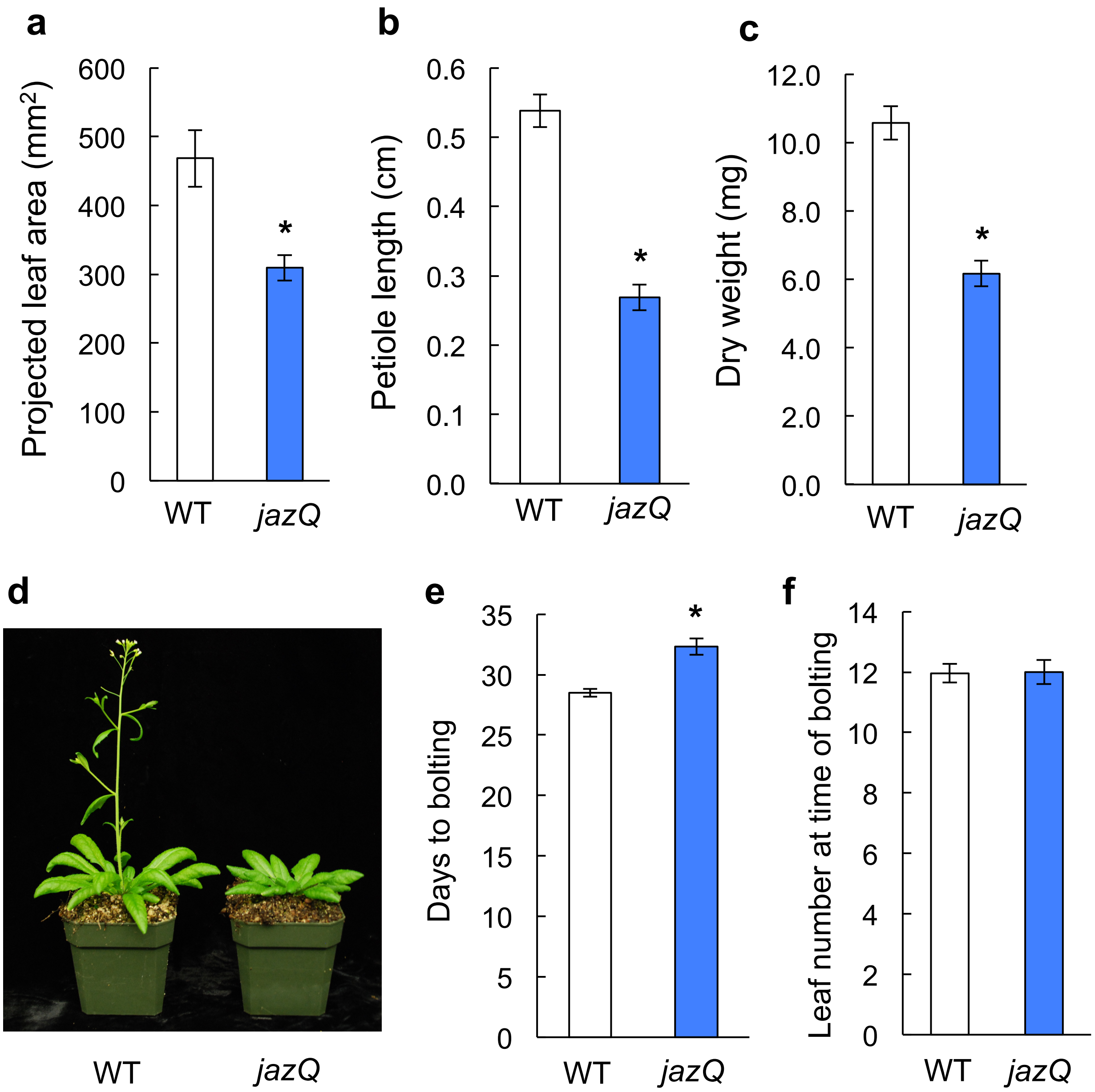

Supplementary Figure 2. Vegetative and reproductive phenotypes of *jazQ*. a, Projected leaf area of WT and *jazQ* plants. **b**, Petiole length of the third true leaf. **c**, Rosette biomass of 21-d-old soil-grown plants. **d**, Photograph of 30-d-old WT and *jazQ* plants. **e**, Bolting time of WT and *jazQ*. **f**, Number of rosette leaves at the time of bolting. Data show the mean ± s.e. of least 20 (panel **a**), 10 (panels **b** and **c**), and 12 (panels **e** and **f**) plants per genotype. Asterisks represent statistical difference between WT and *jazQ* (Student's T-test, $P < 0.05$).

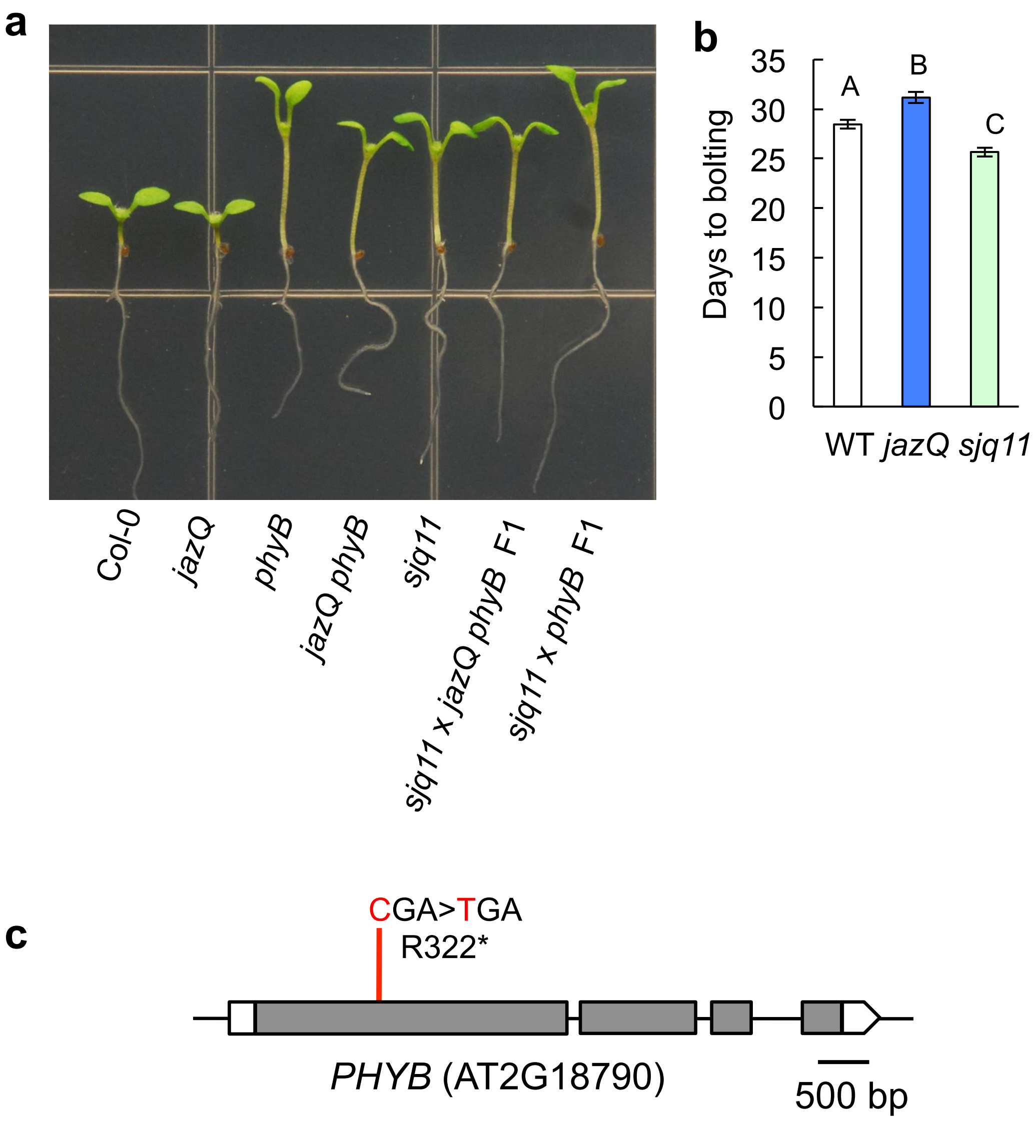

Supplementary Figure 3. The *sjq11* suppressor mutant harbors a null mutation in *PHYB*. **a**, The *sjq11* mutant is allelic to *phyB-9*. Seedlings of the indicated genotype were grown for 5 d (after germination) on MS medium under constant white light (~80 µE m^{-2} s^{-1}). The two seedlings on the far right are F1 plants derived from the indicated genetic cross. **b**, Number of days to bolting. Data show the mean ± standard error (s.e.) of at least 12 independent replicates. Letters indicate statistical differences between genotypes (Tukey HSD-test, $P < 0.05$). **c**, Schematic representation of the *PHYB* gene in *sjq11*. Sequence analysis identified a cytosine (C) to thymine (T) transition that creates a TGA nonsense mutation at the CGA codon for R322. This mutation is predicted to truncate the PHYB apoprotein in the chromophore-binding GAF domain and thus is likely a null mutation.

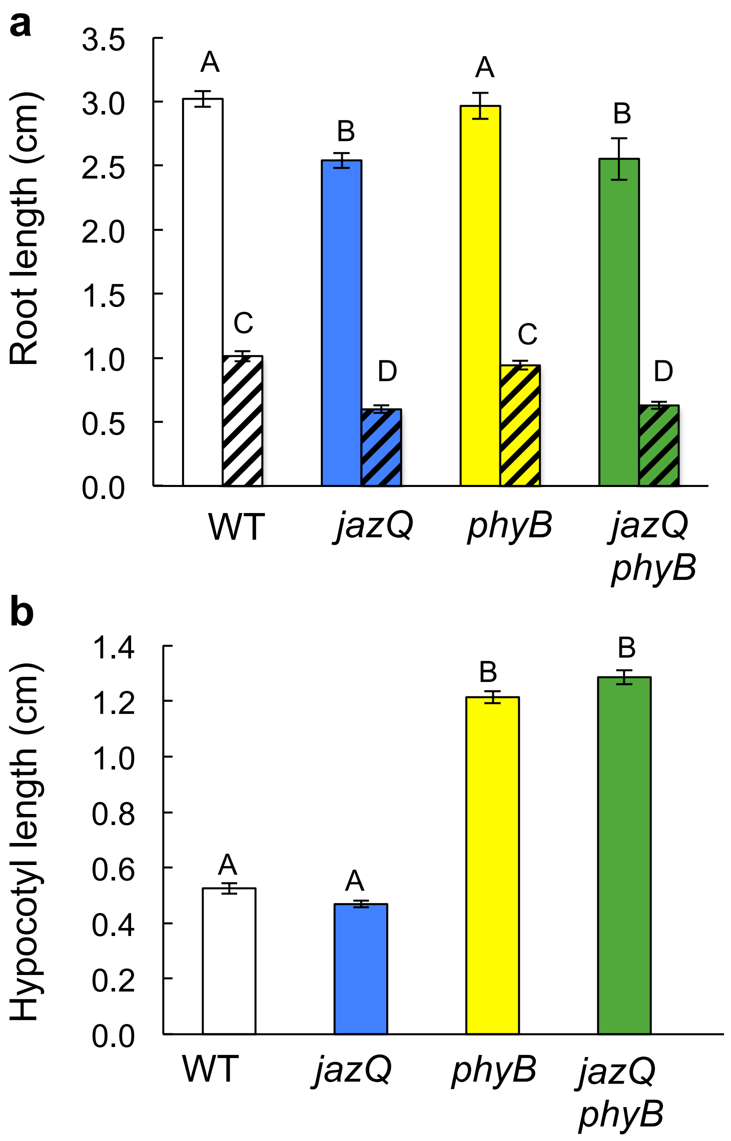

Supplementary Figure 4. *jazQ phyB* plants retain the JA hypersensitivity and red-light insensitivity of *jazQ* and *phyB*, respectively. **a**, Root length of WT, *jazQ*, *phyB,* and *jazQ phyB* seedlings grown for eight d on MS medium supplemented (hatched bar) or not (open bar) with 20 µM MeJA. Data show the mean ± s.e. of at least 12 seedlings per genotype. **b**, Seedlings of the indicated genotype were grown for three d on MS medium in continuous red light at a fluence rate of 25 µE m^{-2} s^{-1}. Data show the mean ± s.e. of hypocotyl length measurements of at least 20 seedlings per genotype. In both panels **a** and **b**, capital letters denote statistical difference at $P < 0.05$ (Tukey HSD-test).

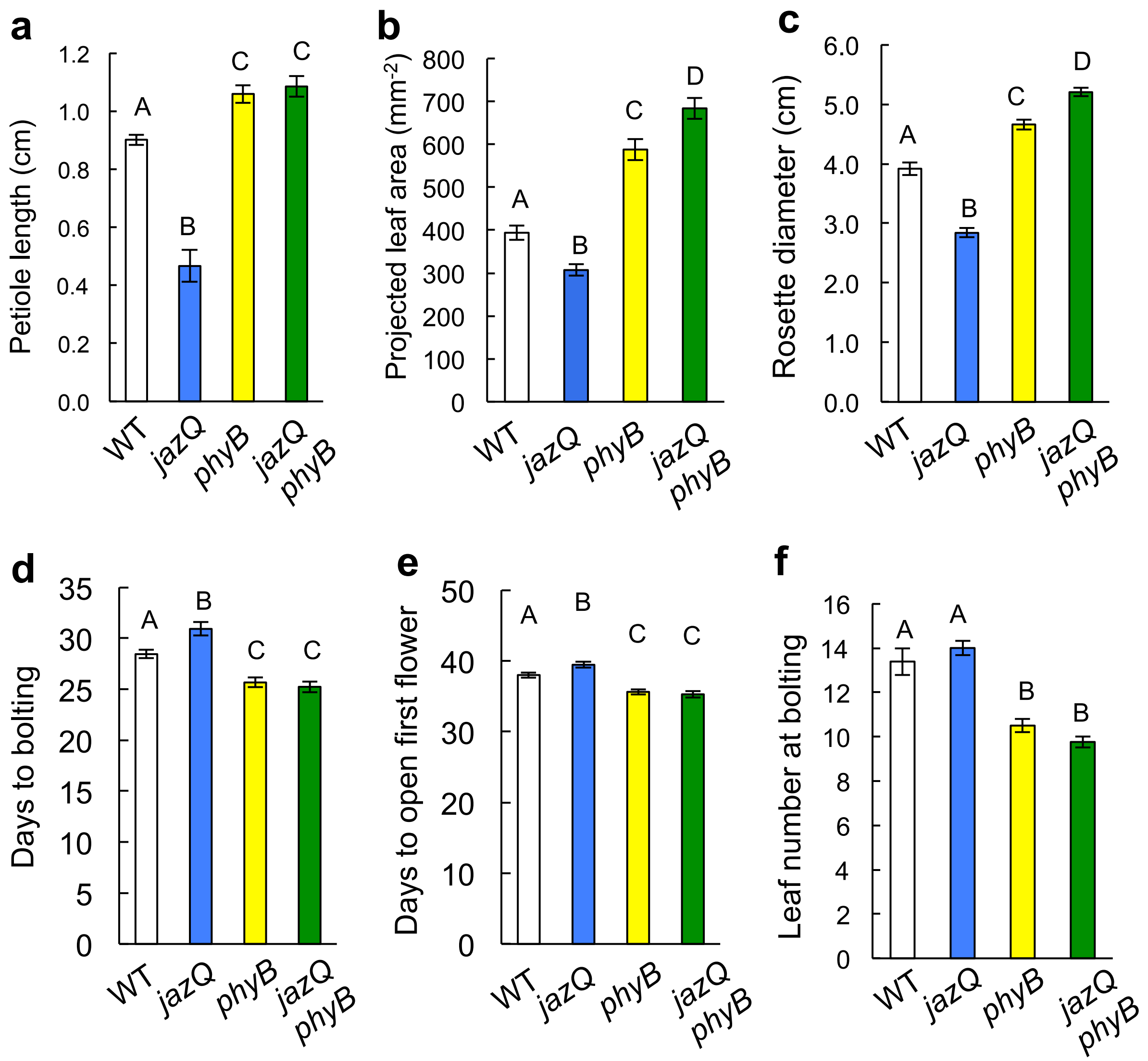

Supplementary Figure 5. *phyB* is epistatic to *jazQ* with respect to several growth parameters. **a**, Petiole length measured on third true leaf of 21-d-old plants. **b**, Projected leaf area of 21-d-old plants. **c**, Rosette diameter of 4-week-old plants. **d**, Number of days to bolting. **e**, Number of days to opening of first flower. **f**, Number of rosette leaves at the time of bolting. Data show the mean ± s.e. of 10 (panel **a**), 20 (panels **b** and **c**), or 12 (panels **d** – **f**) plants per genotype. Capitalized letters indicate statistical differences (Tukey HSD-test, $P < 0.05$).

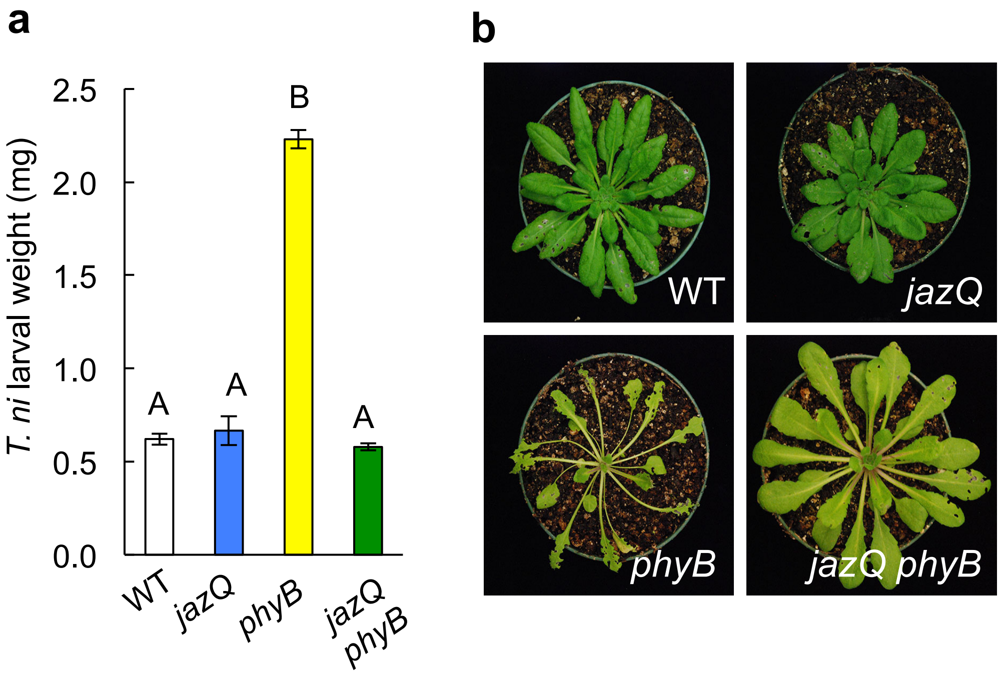

Supplementary Figure 6. *phyB* plants are hyper-susceptible to herbivory by *Trichoplusia ni*. a, *Trichoplusia ni* larval weight after five days of feeding on WT (11 larvae), *jazQ* (10 larvae), *phyB* (36 larvae), and *jazQ phyB* (10 larvae) plants. Feeding trials that included *phyB* mutant plants had to be halted at this time because of complete consumption of *phyB* leaf material. Data represent the mean larval weight ± s.e. of insects recovered from 12 plants per genotype. Letters indicate statistical differences (Tukey HSD-test, $P < 0.05$). Note that due to slow consumption of leaf tissue during the first few days after challenge, the weight of larvae reared on WT, *jazQ*, and *jazQ phyB* plants remain similar during this time period. Significant differences in larval performance on WT and *jazQ*-containing genotypes occurred between the 5- and 10-d post-challenge. **b**, Photograph of representative plants of each genotype at the end of the 5-day feeding assay. The experiment was repeated three times with similar results.

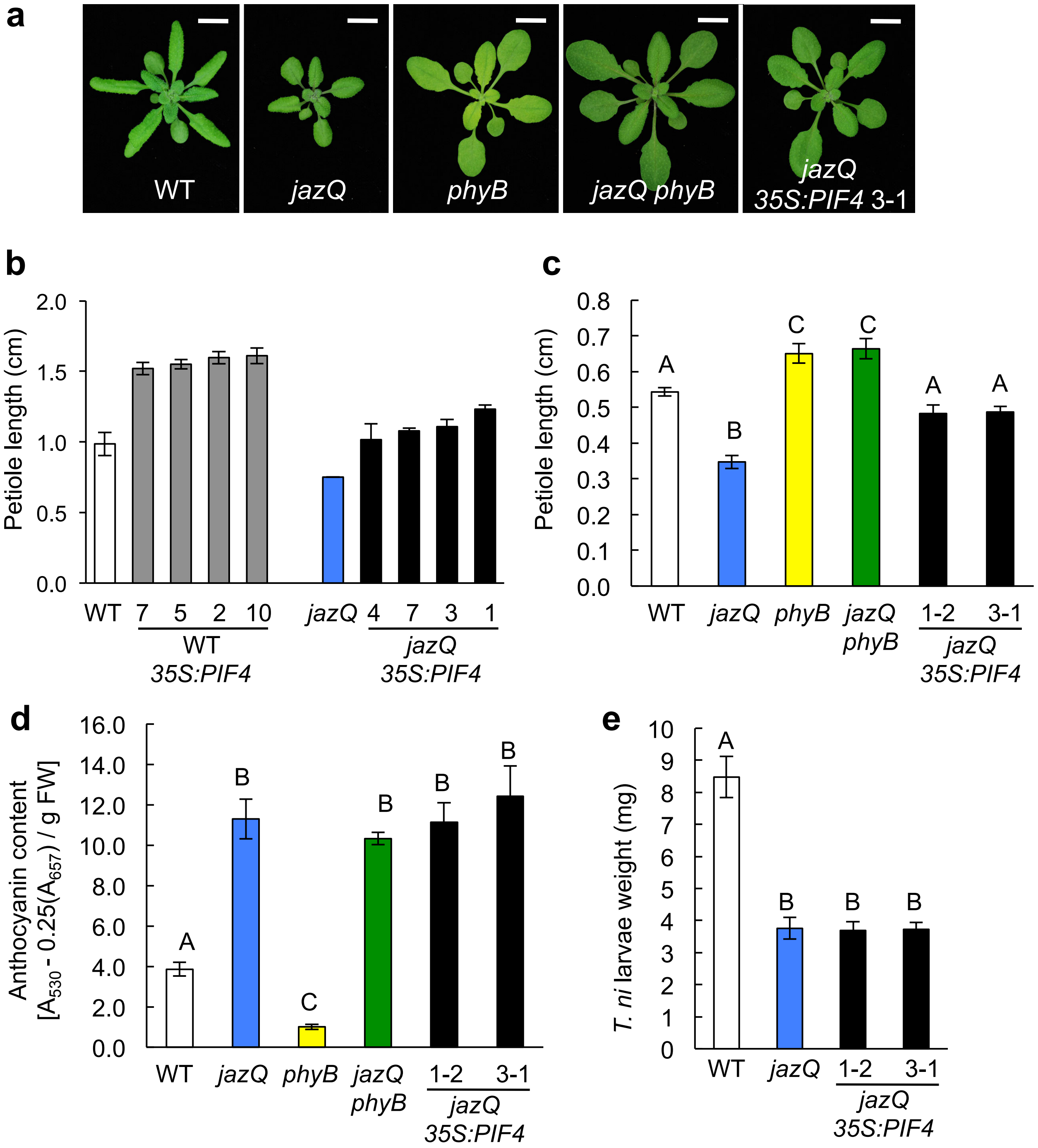

Supplementary Figure 7. Overexpression of *PIF4* in the *jazQ* background leads to partial rescue of growth without compromising defense. a, Photograph of representative 21-d-old plants of the indicated genotype. Two independent T3 lines (#1-2 and #3-1) of *jazQ 35S:PIF4* were characterized but only the latter is pictured. Scale bars = 1 cm. **b**, Petiole length of the third true leaf of independent *jazQ 35S:PIF4* T2 lines (n=6 plants per line). T2 lines #1 and #3 are parents of T3 lines #1-2 and #3-1 described in panels **c**-**e** below. As a control to demonstrate the expected effects of PIF4 overexpression on petiole length, WT Col-0 plants were also transformed with the *35S:PIF4* transgene. Data for four independent T2 lines is shown. **c**, Petiole length of the third true leaf of 21-d-old *jazQ 35S:PIF4* plants compared to WT and *jazQ* (n=10). **d**, Anthocyanin content in petioles of 21-d-old plants of the indicated genotype (n > 10 plants). **e,** Weight of *T. ni* larvae recovered after 10 d feeding on 12 plants per genotype: WT (37 larvae), *jazQ* (31 larvae), *jazQ 35:PIF4* #1-2 (27 larvae), and *jazQ 35:PIF4* #3-1 (25 larvae). Data show the mean ± s.e. Capitalized letters indicate statistical differences (Tukey HSD-test, $P < 0.05$).

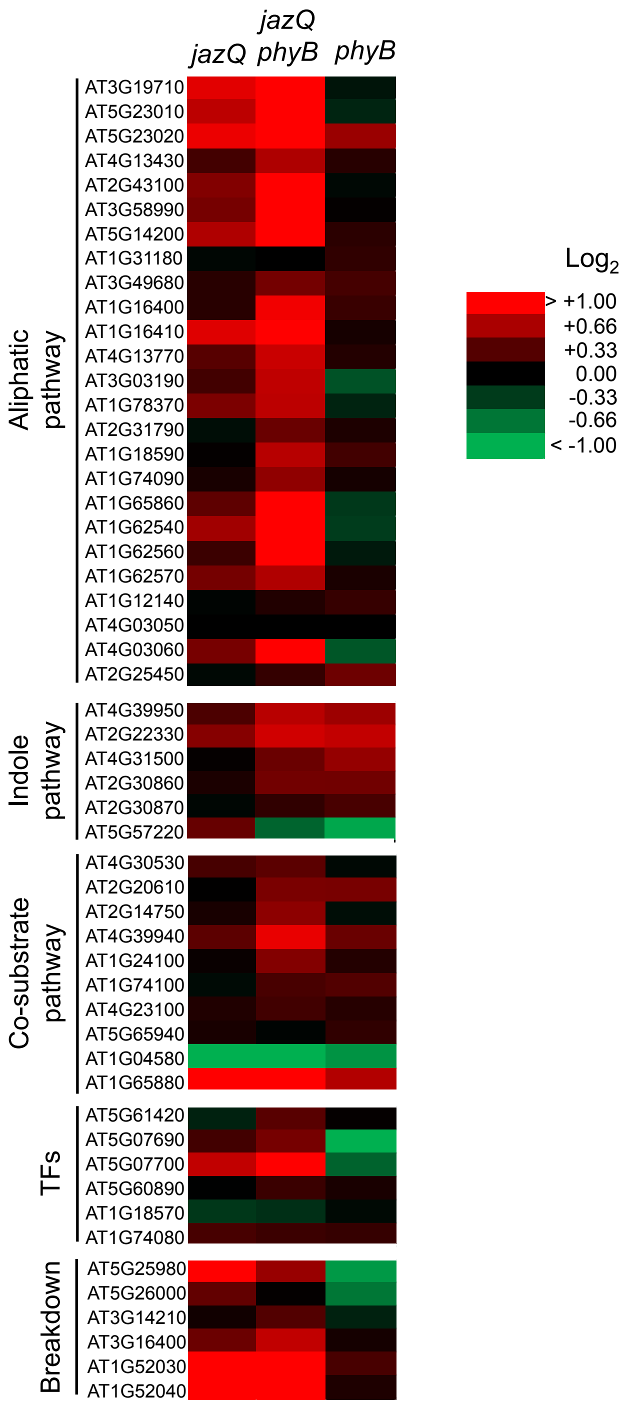

Supplementary Figure 8. Expression of glucosinolate-related genes in various genotypes. Heat map showing the expression level of genes involved in glucosinolate biosynthesis, catabolism, and regulation by transcription factors (TFs). Genes are clustered as described[1,2]. Gene expression levels determined by RNA-seq are represented as fold-change (Log_2) over wild-type Col-0.

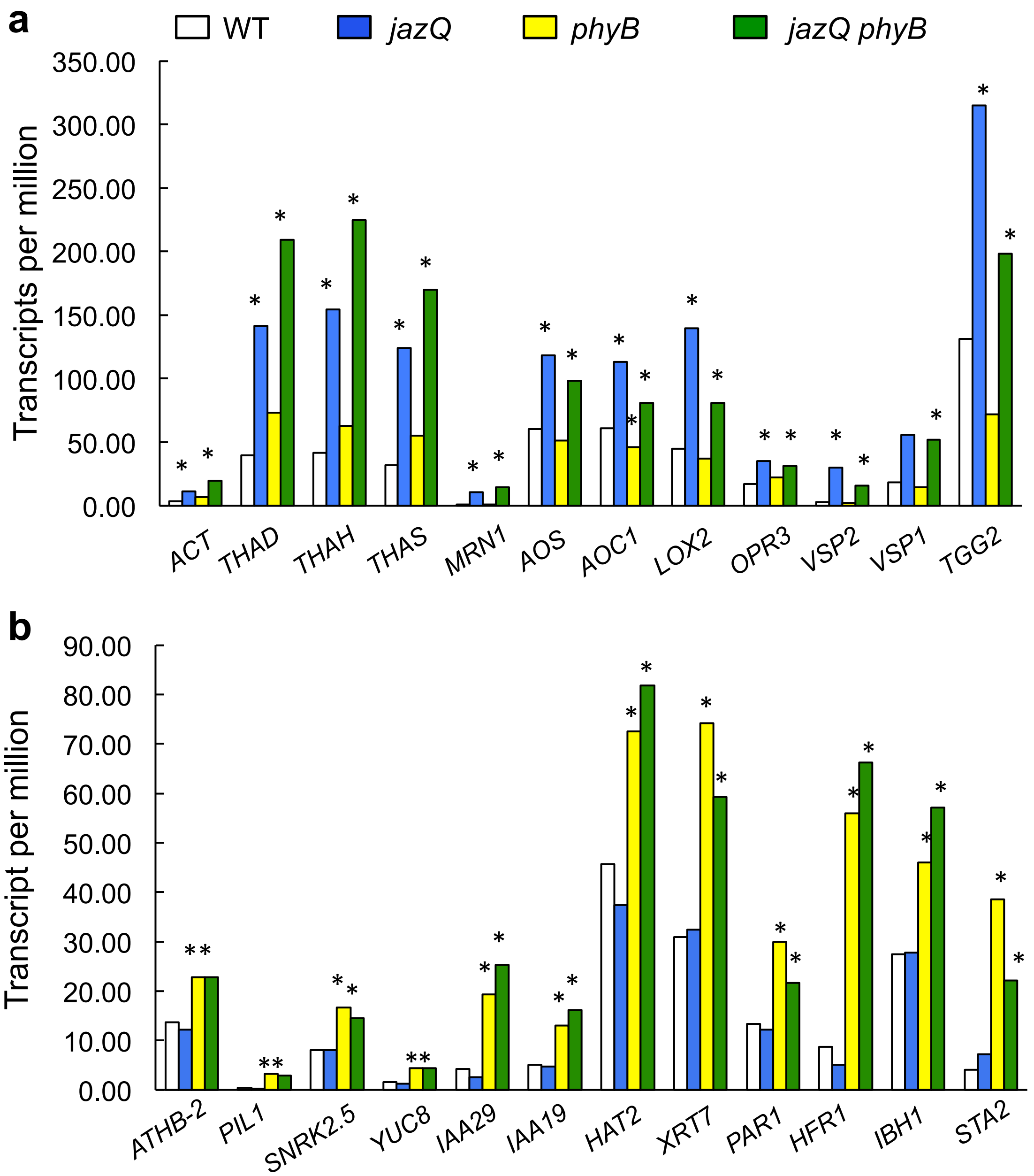

Supplementary Figure 9. Expression of defense- and growth-related genes in wild-type, *jazQ*, *phyB*, and *jazQ phyB* plants. a, Expression level of triterpenoid biosynthesis (*ACT, THAD, THAH, THAS*), JA biosynthesis (*AOS*, *AOC1*, *LOX2*, *OPR3*), and JA-regulated defense (*VSP2*, *VSP1*, *TGG2*) genes as determined by messenger RNA sequencing. **b**, Expression level of genes that are reported to be direct targets of PIF transcription factors. Asterisks denote statistically different expression levels in comparisons to WT (Col-0) according to the DESeq algorithm ($P < 0.01$, using a Benjamini-Hochberg adjusted for multiple testing). Expression variance among the three replicate samples used for each data point is provided in Supplemental Data 1.

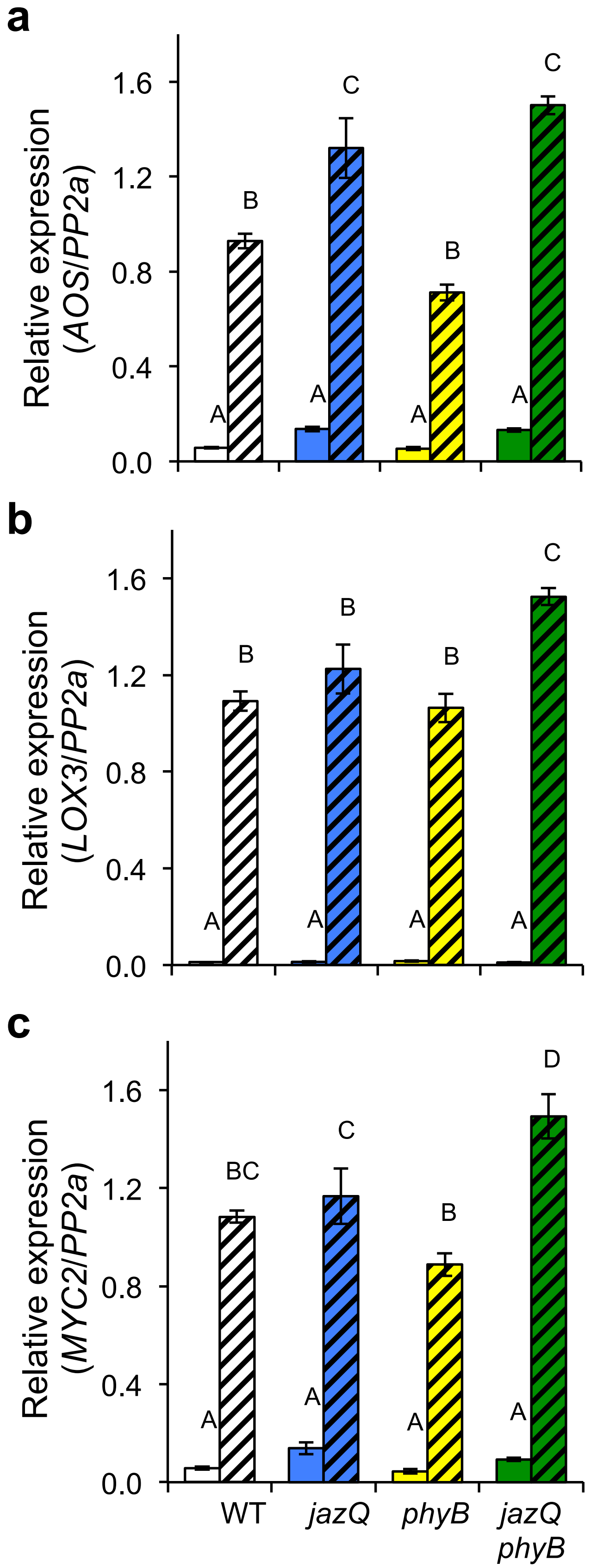

Supplementary Figure 10. Wound-induced expression of early JA-response genes in wild-type and mutant plants. Quantitative PCR was used to determine the expression level of *AOS* (**a**), *LOX3* (**b**) and *MYC2* (**c**) in Col-0, *jazQ*, *phyB*, and *jazQ phyB* plants. RNA isolated from unwounded control leaves (filled bars) or from leaves harvested 1 h after mechanical wounding (hatched bars). Data show the mean ± s.e. and capital letters indicate statistical difference at $P < 0.05$ (Tukey HSD-test). The experiment was repeated twice with similar results.

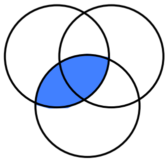

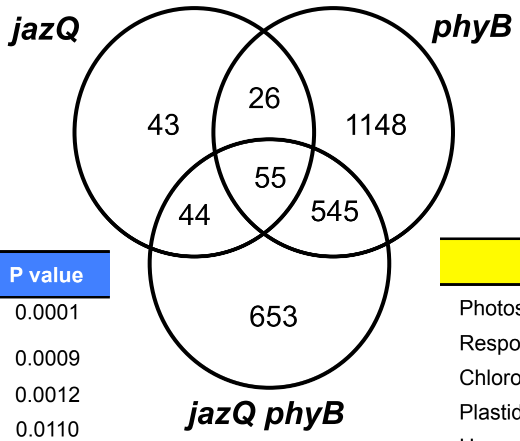

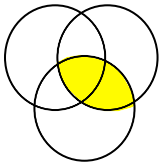

GO description	P value
Response to chitin	0.0001
Regulation of transcription	0.0009
Response to ethylene stimulus	0.0012
Response to salicylic acid stimulus	0.0110
Response to gibberellin stimulus	0.0403

GO description	P value
Photosynthesis	<0.0001
Response to light stimulus	<0.0001
Chlorophyll biosynthetic process	<0.0001
Plastid organization	0.0002
Hexose metabolic process	0.0003
Carbon fixation	0.0003
Glucose catabolic process	0.0045

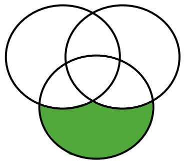

GO description	P value
Response to temperature stimulus	<0.0001
Starch metabolic process	<0.0001
Response to abiotic stimulus	<0.0001
Plastid organization	<0.0001
Circadian rhythm	<0.0001
Embryonic development	<0.0001
Response to oxidative stress	0.0005

Supplementary Figure 11. Gene ontologies for transcripts whose abundance is repressed in *jazQ*, *phyB*, and *jazQ phyB* mutants relative to wild type*.* Venn diagram showing the number of genes that are downregulated in comparisons between WT (Col-0) and each of the three indicated mutants. GO analysis of functional categories was performed with sets of downregulated genes that are shared between *jazQ* and *jazQ phyB* (blue intersect), shared between *phyB* and *jazQ phyB* (yellow intersect), or unique to *jazQ phyB* (green shade) at this expression threshold. Differentially expressed genes were identified from RNA-seq data analyzed with the DESseq algorithm, with significance corresponding to *P* <0.01. See Supplementary Data 1 for detailed expression data and GO analysis.

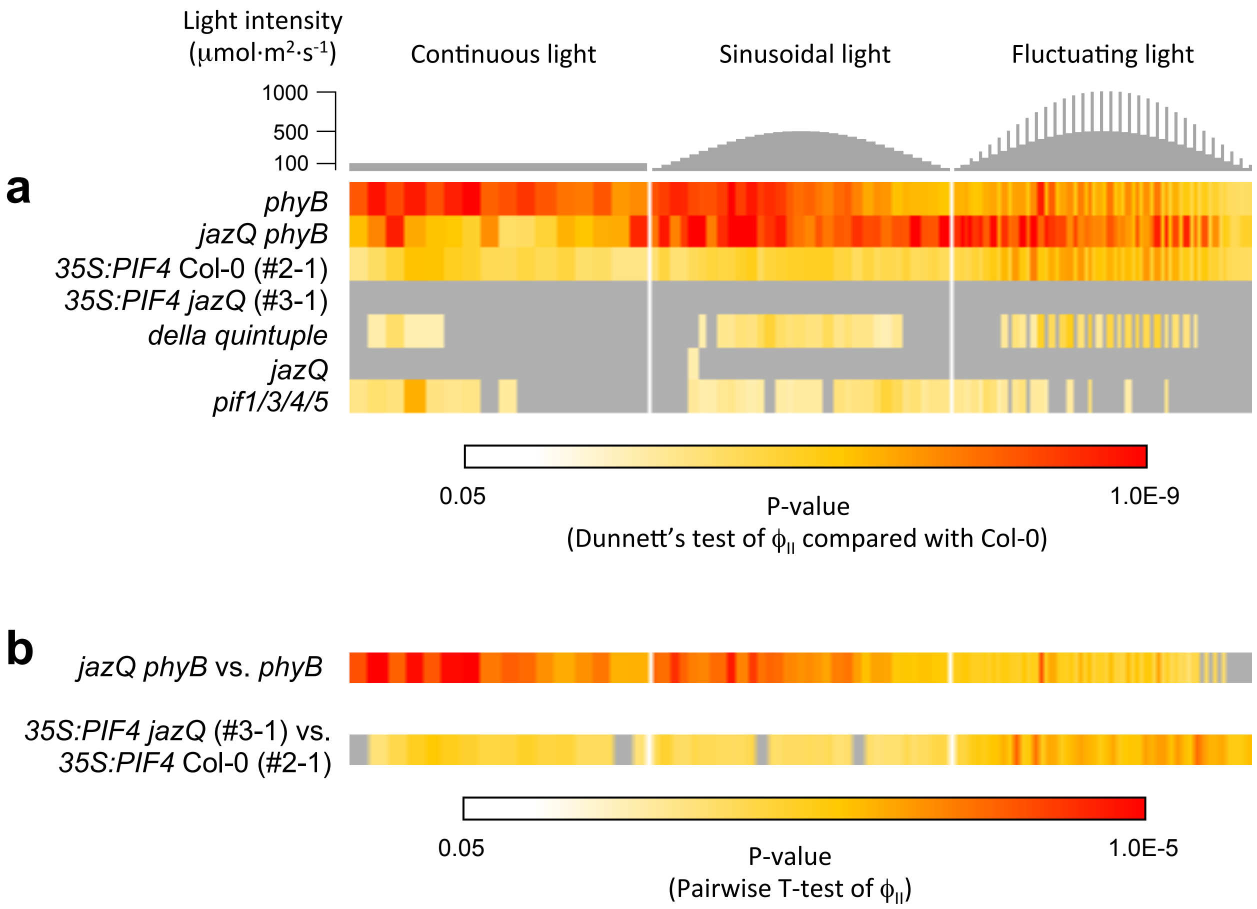

Supplementary Figure 12. Heatmap showing the statistical significance of differences in ϕ_{II}. a, Statistical significance of differences in ϕ_{II} between each mutant and Col-0 at each time point for the data shown in Figure 4, evaluated using Dunnett's test. P-values > 0.05 are shown in grey. **b**, Statistical significance of differences in ϕ_{II} between *jazQ phyB* and *phyB*, and between *35S:PIF4* overexpressors in *jazQ* (line 3-1) and in Col-0 (line 2-1), evaluated using an unpaired, two-tailed t-test. P-values > 0.05 are shown in grey.

Supplementary Table 1. Several PIF4 targets are associated with gene ontology terms for responses to jasmonate and wounding[a].

GO ID	GO Description	p-value[b]
9753	response to jasmonic acid stimulus	2.74E-07
6950	response to stress	1.00E-04
9611	response to wounding	2.94E-04

[a]PIF4 target genes (4,363 genes) were retrieved from Supplementary Table 1 in Oh et al (2012).
[b]Enriched functional categories determined with BiNGO using a hypergeometric test with Benjamini & Hochberg's FDR correction.

Supplementary Table 2. Several PIF4 targets are also reported targets of MYC transcription factors[a].

AGI accession[b]	Gene symbol	Targeted by MYC(s):	Experimental evidence[c]
AT1G17380	*JAZ5*	MYC5	GUS reporter[4]
AT1G17380	*JAZ5*	MYC2/3/4	GUS reporter[5]
AT1G19180	*JAZ1*	MYC5	GUS reporter[4]
AT1G19180	*JAZ1*	MYC2/3/4	GUS reporter[5]
AT1G19180	*JAZ1*	MYC2	Yeast 1-hybrid[6]
AT1G52890	*ANAC019*	MYC2	ChIP-qPCR[7]
AT1G70700	*JAZ9*	MYC5	GUS reporter[4]
AT1G70700	*JAZ9*	MYC2/3/4	GUS reporter[5]
AT1G72450	*JAZ6*	MYC2	ChIP-qPCR[8]
AT1G72450	*JAZ6*	MYC2/3/4/5	GUS reporter[4]
AT1G72450	*JAZ6*	MYC2/3/4	GUS reporter[5]
AT2G18700	*TPS11*	MYC2	GUS reporter, ChIP-qPCR[9]
AT2G22330	*CYP79B3*	MYC2	ChIP-seq[10]
AT2G24210	*TPS10*	MYC2	ChIP-qPCR, EMSA[11]
AT2G25490	*EBF1*	MYC2	ChIP-qPCR, EMSA[12]
AT2G46340	*SPA1*	MYC2	ChIP-PCR, EMSA[13]
AT3G15500	*ANAC055*	MYC2	ChIP-qPCR[7]
AT3G17860	*JAZ3*	MYC2	EMSA[14]
AT5G13170	*SAG29*	MYC2	ChIP-qPCR, LUC reporter[15]

[a]PIF4 target genes (4,363 genes) were retrieved from Supplementary Table 1 in Oh et al.[1].
[b]Among the 4,363 PIF4 targets, the 13 genes listed here are also reported to be targets of MYC transcription factors.
[c]Experimental evidence that the listed target genes are controlled by MYC transcription factors is provided in these publications.

References

1. Sonderby, I. E., Geu-Flores, F. & Halkier, B. A. Biosynthesis of glucosinolates--gene discovery and beyond. *Trends Plant Sci* **15**, 283-290, (2010).
2. Schweizer, F. *et al.* Arabidopsis basic helix-loop-helix transcription factors MYC2, MYC3, and MYC4 regulate glucosinolate biosynthesis, insect performance, and feeding behavior. *Plant Cell* **25**, 3117-3132 (2013).
3. Oh, E., Zhu, J. Y. & Wang, Z. Y. Interaction between BZR1 and PIF4 integrates brassinosteroid and environmental responses. *Nat Cell Biol* **14**, 802-809 (2012).
4. Figueroa, P. & Browse, J. Male sterility in Arabidopsis induced by overexpression of a MYC5-SRDX chimeric repressor. *Plant J* **81**, 849-860 (2015).
5. Niu, Y., Figueroa, P. & Browse J. Characterization of JAZ-interacting bHLH transcription factors that regulate jasmonate responses in Arabidopsis. *J Exp Bot* **62**, 2143-2154 (2011).

6. Pauwels, L. & Goossens, A. Fine-tuning of early events in the jasmonate response. *Plant Signal Behav* **3**, 846-847.
7. Zheng, X. Y. et al. Coronatine promotes *Pseudomonas syringae* virulence in plants by activating a signaling cascade that inhibits salicylic acid accumulation. *Cell Host Microbe* **11**, 587-596.
8. Chen, R. *et al*. The Arabidopsis mediator subunit MED25 differentially regulates jasmonate and abscisic acid signaling through interacting with the MYC2 and ABI5 transcription factors. *Plant Cell* **24**, 2898-2916 (2012).
9. Hong, G. J. *et al*. Arabidopsis MYC2 interacts with DELLA proteins in regulating sesquiterpene synthase gene expression. *Plant Cell* **24**, 2635-2648 (2012).
10. Schweizer, F. *et al*. Arabidopsis basic helix-loop-helix transcription factors MYC2, MYC3, and MYC4 regulate glucosinolate biosynthesis, insect performance, and feeding behavior. *Plant Cell* **25**, 3117-3132 (2013).
11. Li, R. *et al*. Virulence factors of geminivirus interact with MYC2 to subvert plant resistance and promote vector performance. *Plant Cell* **26**, 4991-5008 (2014).
12. Zhang, X. *et al*. Jasmonate-activated MYC2 represses ETHYLENE INSENSITIVE3 activity to antagonize ethylene-promoted apical hook formation in Arabidopsis. *Plant Cell* **26**, 1105-1117.
13. Gangappa, S. N., Prasad, V. B. & Chattopadhyay, S. Functional interconnection of MYC2 and SPA1 in the photomorphogenic seedling development of Arabidopsis. *Plant Physiol* **154**, 1210-1219 (2010).
14. Chini, A. *et al*. The JAZ family of repressors is the missing link in jasmonate signalling. *Nature* **448**, 666-671 (2007).
15. Qi, T. *et al*. Regulation of jasmonate-induced leaf senescence by antagonism between bHLH subgroup IIIe and IIId factors in Arabidopsis. *Plant Cell* **27**, 1634-1649.
