# Supplementary material for: Rewiring of jasmonate and phytochrome B signalling uncouples plant growth-defense tradeoffs
Source: Nat Commun. 2016 Aug 30;7:12570. doi: 10.1038/ncomms12570 (PMC5155487; doi:10.1038/ncomms12570)
Supplement: Peer Review File [file ncomms12570-s3.pdf]

Editorial Note: This manuscript has been previously reviewed at another journal that is not operating a transparent peer review scheme. This document does not contain reviewer comments and rebuttal letters as the version considered at *Nature Communications* was not sent for further formal peer review.
